# Temperature elevations can induce switches to homoclinic action potentials that alter neural encoding and synchronization

**DOI:** 10.1038/s41467-022-31195-6

**Published:** 2022-07-08

**Authors:** Janina Hesse, Jan-Hendrik Schleimer, Nikolaus Maier, Dietmar Schmitz, Susanne Schreiber

**Affiliations:** 1 Institute for Theoretical Biology, Humboldt-Universität zu Berlin, Berlin, Germany; 2 Bernstein Center for Computational Neuroscience, Berlin, Germany; 3 Neuroscience Research Center - Charité-Universitätsmedizin Berlin, Corporate member of Freie Universität Berlin, Humboldt-Universität zu Berlin, and Berlin Institute of Health, German Center for Neurodegenerative Diseases (DZNE) Berlin, Berlin, 10117 Germany; 4 Max-Delbrück-Centrum (MDC) for Molecular Medicine, Berlin, 13125 Germany; 5 Present Address: Institute for Systems Medicine, Department of Human Medicine, MSH Medical School Hamburg—University of Applied Sciences and Medical University, Hamburg, 20457 Germany; 6 Present Address: Charité-Universitätsmedizin Berlin, Corporate member of Freie Universität Berlin, Humboldt-Universität zu Berlin, and Berlin Institute of Health, Berlin, Germany

**Keywords:** Dynamical systems, Network models

## Abstract

Almost seventy years after the discovery of the mechanisms of action potential generation, some aspects of their computational consequences are still not fully understood. Based on mathematical modeling, we here explore a type of action potential dynamics – arising from a saddle-node homoclinic orbit bifurcation - that so far has received little attention. We show that this type of dynamics is to be expected by specific changes in common physiological parameters, like an elevation of temperature. Moreover, we demonstrate that it favours synchronization patterns in networks – a feature that becomes particularly prominent when system parameters change such that homoclinic spiking is induced. Supported by in-vitro hallmarks for homoclinic spikes in the rodent brain, we hypothesize that the prevalence of homoclinic spikes in the brain may be underestimated and provide a missing link between the impact of biophysical parameters on abrupt transitions between asynchronous and synchronous states of electrical activity in the brain.

## Abstract (web summary)

The intrinsic dynamics of neurons, in particular the generation action potentials, can impact neural network states and processes of encoding information. The authors demonstrate how the elevation of temperature induces a type of action potential dynamics that favors synchronization patterns in neural networks.

## Introduction

Action potentials are the central information unit in our brain^1,2^. Despite the large complexity of the underlying composition of ion channels—all-or-none spike generation in regularly firing neurons comes along in only three different dynamical types, defined by qualitative features of the transition that induces firing at threshold^3–5^. While two of these types have been extensively considered and seemingly sufficed to characterize the behavior of many experimentally observed neurons^6,7^, including the class 1 and 2 excitability described in ref. ^8^, the prevalence and computational potential of the remaining type has been ignored. We argue that this neglect is unjustified, because these *homoclinic* action potentials (as we term them here) can be easily induced in common conductance-based neuron models and strongly impact the behavior of the embedding network even when synaptic connections remain unchanged.

**Fig. 1:**
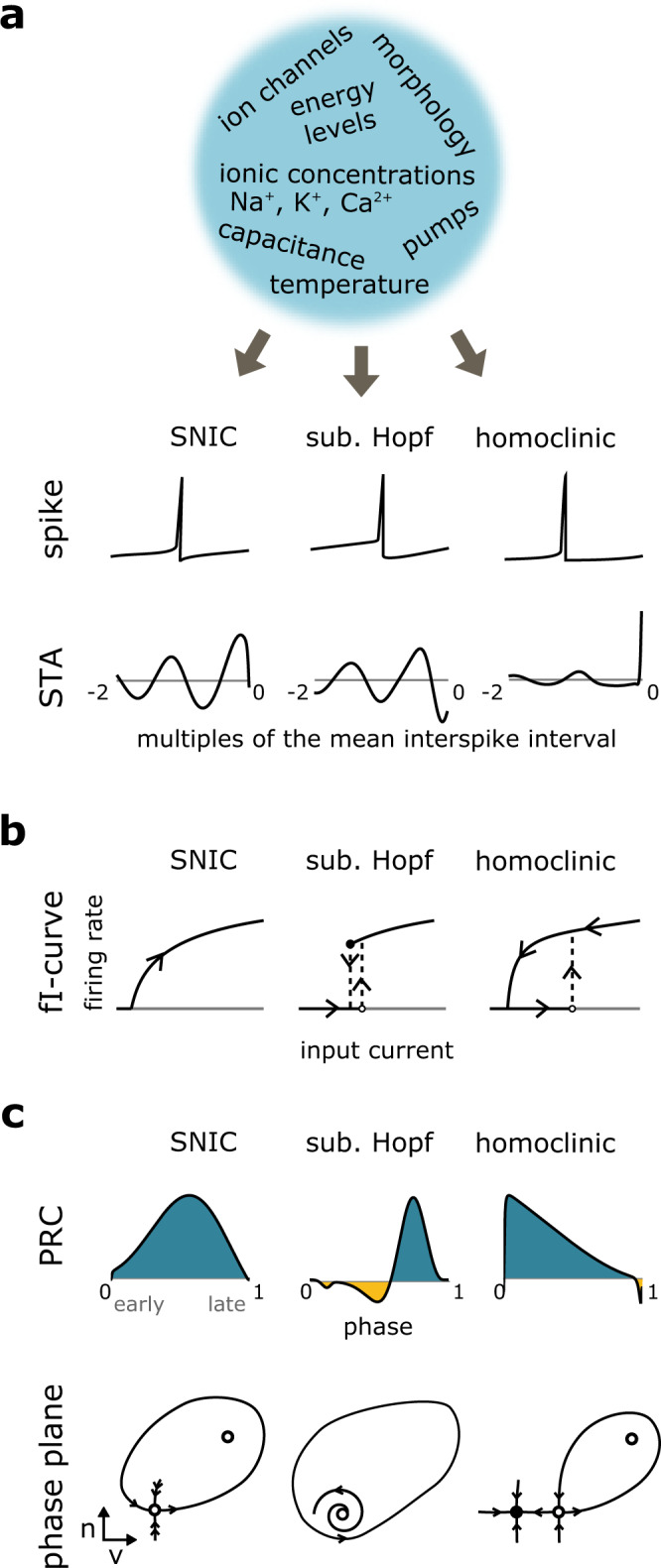
Action-potential dynamics of regularly firing cells come in three mathematical types with different spike onset bifurcations, saddle node on invariant cycle (SNIC), saddle-homoclinic orbit (HOM), and subcritical Hopf bifurcation. **a** Biophysical properties set the spike onset dynamics, including the spike-triggered averages (STAs) close to the threshold. **b** The three spike onset dynamics differ in their firing-rate-input curves (f–I curves), **c** typical phase-response curves (PRCs, top), and phase planes (bottom); closed circles denote stable fixed points, open circles unstable fixed points, and half-filled circles a saddle node. Arrows indicate the direction of the dynamics.

The dynamics of action potentials are shaped by the biophysical properties of the neurons that generate these pulses (Fig. 1). The qualitative features of action potentials, however, depend on the dynamical transition from the subthreshold range to a regular firing regime and thus on the details of how spiking is induced at the threshold. Mathematically, this transition happens at a bifurcation, i.e., a point of qualitative change in a system’s dynamics. For the induction of regular firing with nonvanishing action-potential amplitude, three such onset bifurcations can occur: the so-called Saddle-Node-on-Invariant Cycle bifurcation (SNIC), the subcritical Hopf bifurcation, and the saddle-HOMoclinic orbi*t* bifurcation (HOM). The first two give rise to what we term the “classical” action-potential types, the latter yields homoclinic action potentials with the interesting properties discussed in this study.

**Fig. 2:**
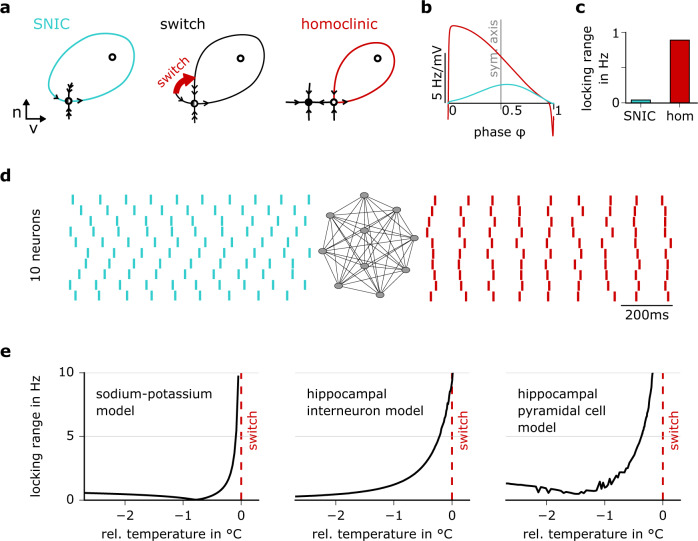
Network synchronization depends on the type of cell-intrinsic dynamics. **a** Phase portraits of the saddle-node-on-invariant cycle (SNIC) and homoclinic dynamics illustrate the co-existence of a stable resting (full circle) and spiking attractor in the homoclinic case, once the limit cycle detaches from the saddle fixed point (red homoclinic orbit in the right panel). **b** Phase-response curves (PRCs) of model neurons with a SNIC spike onset (blue), and a homoclinic spike onset (red). **c** The asymmetric PRC of the homoclinic neuron (compare **b**, red trace) results in a larger locking range than the symmetric PRC of the SNIC neuron (compare **b**, blue trace). **d** Spiking responses of a fully coupled inhibitory network of model neurons with SNIC (blue) or homoclinic (red) spike onset. Distinct network synchronization results entirely from differences in cell-intrinsic dynamics. **e** A temperature rise changes firing from SNIC to homoclinic in diverse conductance-based models. Locking range as a function of temperature (relative to the model’s critical transition temperature from SNIC to homoclinic spiking).

The qualitative properties of action potentials depend on the underlying bifurcation. For example, the two classical bifurcation types differ in the onset firing frequency at the threshold, which can be arbitrarily low (SNIC) or immediately jumps to larger values, excluding very low rates (subcritical Hopf), see Fig. 1b. Both types also differ in their encoding, like the spike-triggered average stimulus waveform (STA), their tendency to give rise to subthreshold oscillations of the membrane potential, or their contribution to a synchronization of the embedding network^3–5^. For homoclinic spike generation, not much is known in the context of neural processing. Homoclinic spikers might be particularly interesting for the processing of high frequencies because in contrast to the spike-triggered averages of Hopf and SNIC spikers, which show oscillations with a period around the mean interspike interval, the STA of a homoclinic spiker shows a fast transient that contains high frequencies (Fig. 1a). While the underlying mathematical bifurcation is well-known and explored in other dynamical systems across diverse fields (from lasers and semiconductors to predator-prey systems^9–11^), its role in the brain has been surprisingly unattended. Mathematically, a prerequisite of homoclinic spikes is the existence of a saddle fixed point. The saddle is marked by attractive dynamics in one direction and repulsive dynamics in another. A trajectory that leaves the saddle along a repulsive direction and loops around to return to the saddle along an attractive direction forms a homoclinic orbit (Fig. 2a, right)^5^. The homoclinic orbit corresponds to homoclinic spiking in the limit of zero firing rate, and repetitive homoclinic spiking occurs when the homoclinic orbit detaches from the saddle to form a stable limit cycle^5^. The global nature of the HOM bifurcation may have rendered its mathematical exploration more difficult in comparison to the other two local bifurcations. Equally if not more important, however, is the impression established early on that the two classical bifurcations seemingly suffice to capture the two physiological excitability classes described by Hodgkin, SNIC falling into class 1 and the subcritical Hopf falling into class 2. In contrast, the homoclinic bifurcation did not allow such a clear distinction, because its somewhat more complex properties result in a double nature with respect to the most prominent distinction between Hodgkin’s class 1 and 2 excitability: When gradually increasing an input (starting in the subthreshold range), neurons with HOM dynamics present themselves as class 2, exhibiting a jump in frequency at the threshold. When starting from a regular firing mode, gradually decreasing the input, HOM neurons present as class 1, exhibiting arbitrarily low rates before the smooth transition to rest (Fig. 1b). This property makes them easy to overlook, given that they readily appear to match one of the two classical types in the most commonly used stimulus protocols for f–I characterization^5^.

The most interesting property of homoclinic action potentials, however, is not the mixture of class 1 and 2 excitability. As we demonstrate here in conductance-based neuron models, homoclinic action potentials possess a characteristic that distinguishes their dynamics from both classical excitability classes and results in novel properties that are likely to play a role for the information transferred by such action potentials as well as the effect of cells with these spike dynamics on the dynamics of the embedding network. We argue that the temporal sensitivity of neurons with homoclinic action-potential dynamics is distinct and favors synchronization patterns of networks (including in-phase synchronization across all cells as well as so-called frustrated network states with diverse combinations of fixed phase relations between cells; both are explained in more detail below)^12^. As the change in temporal sensitivity occurs abruptly when leaving the classical SNIC spiking regime, the induction of homoclinic firing can trigger synchronization patterns by comparatively small changes in parameters, like a temperature elevation of only 2 °C, which, for example, for inhibitory networks results in strong network rhythms. While here exemplified by modifications in temperature, homoclinic action potentials can be easily induced by changes in other physiological parameters. We use our model predictions to complement our analysis with in vitro experimental hallmarks suggesting the possibility of homoclinic spikes at elevated temperatures in hippocampal CA1 pyramidal cells.

## Results

The intrinsic dynamics of neurons defines their computational properties^13,14^. For regularly firing neurons, an important part of their dynamics is reflected in the neuron’s temporal sensitivity to inputs, captured by the so-called phase-response curve (PRC). At depolarization levels above the threshold, the PRC quantifies how strongly a small, transient input affects the timing of the next spike. The resulting delay or advance in the next spike’s timing depends on the specific timing of the input during the interspike interval (i.e., early or late)^4,15,16^. Interestingly, the PRC is, on the one hand, characteristic for the bifurcation that underlies action-potential generation. On the other hand, due to the relevance of temporal relationships for neuronal interactions, it also predicts the collective temporal behavior of neurons when they are weakly connected in a network^17^.

### The PRC is specific to each spike dynamical type

At the bifurcation (i.e., at threshold) the PRC tested with infinitesimal input amplitude has a canonical form that is specific to the dynamical type (Fig. 1c). The PRC of the classical SNIC has a characteristic one-minus-cosine shape that peaks at the middle of the interspike interval. Subcritical Hopf bifurcations are marked by PRCs that exhibit negative and positive parts, representing both advances and delays. In the homoclinic case, spike generation comes along with a PRC that gives rise to large advances of the next spike when the input occurs right after the last action potential. This effect decreases monotonically over the interspike interval. In other words, the temporal sensitivity to inputs is particularly pronounced in a period where neurons are commonly considered refractory. Refractoriness is not lost per se; an input does not trigger an immediate action potential. Yet depolarizing inputs arriving early in the firing cycle have the largest influence on the timing of the following spike.

### PRC asymmetry impacts network synchronization patterns

When comparing the PRCs across the three dynamics, there are apparent differences in the symmetries of the curves. The PRC of the classical SNIC is symmetric with respect to phase 0 (note that the PRC is a periodic function, it repeats below zero; for easier read-off, symmetry can also be evaluated against a vertical axis centered at phase 0.5). In contrast, the PRCs of the subcritical Hopf and the HOM are not symmetric. The PRC of the homoclinic case is particularly asymmetric because the timing sensitivity before and after a spike (i.e., to the left and right of 0) differ strongly. Interestingly, the PRC asymmetry is a property that is known to predict the population activity of neurons in fully coupled networks^18^. Under the assumption of fast synaptic transmission, a fixed phase relationship between the firing of neurons in the network can be expected if the asymmetry of the PRC is strong^18^. In other words, the more asymmetric the PRC shape, the stronger can be the phase locking of these neurons in the network.

This fact provides an intuition for why networks with identical connectivity can exhibit distinct behaviors for neurons of different spike generation types: Inhibitory coupling of neurons with weakly versus strongly asymmetric PRCs (Fig. 2b, c) leads to distinct synchronization patterns in the mathematical simulation (Fig. 2d). The resulting population activity of the two networks differs, although the network topology is the same and neurons even fire action potentials at identical rates. For fast synaptic transmission, PRC asymmetry correlates with the so-called locking range. In the limit of instantaneous transmission, the locking range can be derived from the odd (i.e., asymmetric) part of the PRC as the difference between the latter’s maximum and minimum (because a frequency detuning between coupled oscillators in this range still leads to stable phase relations between oscillators, see “Methods” for further details). In other words, the locking range here scales with PRC asymmetry and directly quantifies the range of frequencies across which a fixed phase locking between two coupled neurons can be observed. Note that units of the locking range are given in Hz; units of the PRC are Hz per unit of stimulation (like Hz/pA or Hz/mV). Importantly, PRC asymmetry is a property of single-cell dynamics, yet because of its relation to the locking range in the all-to-all coupled network scenario with fast synaptic transmission (Fig. 2d), it causally relates to properties of network synchronization patterns.

### A direct transition from the classical SNIC to homoclinic spikes

A neuron’s computational properties depend on the specific composition of cellular parameters. The latter live in a high-dimensional space, as many biophysical parameters influence electrical activity. When comparing cells with the two classical dynamics, SNIC and subcritical Hopf, it can be assumed that they differ significantly at least in some dimensions of cellular parameters, as, topologically, the two dynamics are located in separate regions of the parameter space that are not neighboring. The situation is different for the relation between the classical SNIC and homoclinic dynamics. These are direct neighbors and, consequently, small changes in one parameter can suffice to tune a neuron’s SNIC action potentials into homoclinic ones when the neuron is placed close to the transition border. In fact, we already know that three parameters can cause such a switch in conductance-based neuron models: membrane capacitance^19^, membrane leak (as can be deduced from the bifurcation analysis in ref. ^20^), and extracellular potassium concentration^21^. As we demonstrate next, the temperature is yet another parameter that can tune spiking dynamics.

Temperature can be introduced in conductance-based neuron models via a three-fold effect (on gating speed, gating peak conductance, and reversal potentials, in order of decreasing impact)^22^. Bifurcation analysis shows that in a neuron model with classical SNIC dynamics, a temperature increase eventually switches spiking dynamics to the homoclinic type. Figure 2e illustrates this finding in three representative and well-established mathematical neuron models, the vertical red line marking the transition point. For two-dimensional conductance-based models, like the sodium–potassium model in Fig. 2e, this switch can even be mathematically proven to exist in any model of SNIC dynamics whose temperature is increased (in analogy to the proof provided by Hesse and colleagues^19^). Focussing on the relationship between the switching point and the locking range, the rate of change in the latter accelerates towards the transition (i.e., the slope is particularly steep), favoring the locking of spikes when approaching homoclinic dynamics. This property arises from the fact that the system suddenly switches from a slow trajectory (SNIC case) to the fast manifold (homoclinic case), see Fig. 2a. Switching the spike downstroke to the fast manifold effectively halves not only the time spent around the saddle node, but also the PRC; accordingly, the homoclinic PRC at the switch corresponds to the second half of the SNIC PRC, see “Methods”, yielding an intuition for why the PRC changes from symmetric to asymmetric (when switching from SNIC to HOM) and, consequently, why the locking range strongly increases. Indeed, the major part of the boost in the locking range occurs within a narrow temperature interval of only 1 °C. Due to the strong effect on PRC asymmetry (and therefore locking range), the switch in dynamical type can be expected to be accompanied by a stark increase in the ability to produce synchronized network patterns when approaching the transition point (Fig. 2d), as discussed further down.

Mathematically, the trend of an increasing asymmetry and locking range continues into the homoclinic regime. We note, however, that the described switch in manifolds results in an instability of the numerical continuation, not permitting us to portray the curve beyond the switching point, see “Methods” for an approximate numerical method.

### The two-dimensional bifurcation structure

**Fig. 3:**
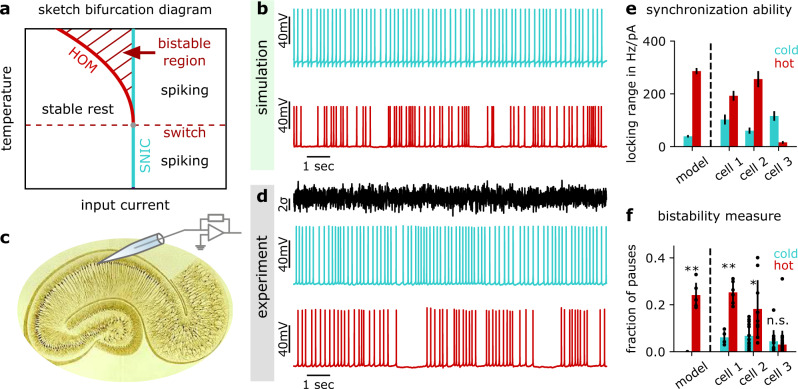
Experimental hallmarks of the critical transition induced by a temperature rise. **a** The temperature-dependent bifurcation diagram shows a region of bistability above the critical temperature (shaded area). **b** Neuron model responses to a noisy current for SNIC and homoclinic spiking (blue and red traces, respectively). At the warmer temperature (homoclinic firing), the model exhibits a characteristic pattern of intermittently interrupted firing arising from the bistability. **c** Schematic of the recording site. **d** Experimental voltage traces in a hippocampal CA1 neuron at two different temperatures (blue 32.3 °C, red 37.7 °C) when stimulated with a noisy current (top trace). Indeed, at the elevated temperature, irregular, interrupted firing is induced. **e** Locking range as an indicator of synchronization ability at the colder and warmer temperature (blue and red, respectively for three experimental cells and the model simulation; model temperatures −2.6 °C and +0.9 °C relative to the switching temperature; cell 1: 32.3 °C versus 37.7 °C, cell 2: 31.9 °C versus 40.0 °C, cell 3: 32.2 versus 40.0 °C; mean across all spikes and +/− SD from bootstrapping, 200 samples with half of the spikes). **f** Fraction of pauses as an indicator of bistability at the colder and warmer temperature obtained for the same dataset as in (**e**), including the model cell (mean and +/− SD across snippets). Two example cells showed synchronization and bistability characteristics consistent with a switch from SNIC to homoclinic dynamics. Significance was evaluated as difference in median by a two-sided Mann–Whitney *U* test with test statistic *U* (model: *P* = 0.0079, *U* = 15, *N*_*cold*_ = 5, *N*_*hot*_ = 5; cell 1: *P* = 0.0095, *U* = 10, *N*_*cold*_ = 4, *N*_*hot*_ = 6; cell 2: *P* = 0.0232, *U* = 115, *N*_*cold*_ = 13, *N*_*hot*_ = 9; cell 3: *P* = 0.1483, *U* = 259.5, *N*_*cold*_ = 11, *N*_*hot*_ = 27).

As we saw above, each spike-generating bifurcation type is associated with unique properties that affect the temporal sensitivity via the PRC. Moreover, each bifurcation type is also marked by a specific bifurcation structure that takes the presence of one or several of so-called attractor states into account (like resting states, mathematically corresponding to fixed points, or regular spiking, mathematically corresponding to a limit cycle). Changes in bifurcation structure at transition points between different types of spike-generating bifurcations affect characteristic features of neuronal dynamics. Specifically, when switching from the classical SNIC to homoclinic dynamics, neurons exhibit a novel property: their voltage becomes bistable. This means that at a given level of stimulation, such as constant input current, two different voltage dynamics can arise. Depending on the initial conditions (like the initial voltage value when a stimulation was started), homoclinic neuronal dynamics can either be regularly spiking (i.e., settle onto a limit cycle in mathematical terms) or, alternatively, converge towards a fixed voltage value (like in the subthreshold regime). For homoclinic spike generation, this bistability is present for inputs directly above threshold, because the saddle-node bifurcation, at which the stable resting state is destroyed, happens at a higher input current than the creation of the limit cycle from the saddle (Fig. 2a). Such bistable dynamics do not exist in the classical SNIC case. For constant inputs below threshold, SNIC dynamics converge to a fixed voltage level; for constant inputs above threshold (yet far from the excitation block), the dynamics converge towards regular firing (i.e., a limit cycle), irrespective of initial conditions. The structure of the possible bifurcations when increasing the temperature of a SNIC neuron is summarized in a two-dimensional bifurcation diagram (spanned by the dimension of input current and temperature, Fig. 3a). The bistable zone co-occurring with homoclinic dynamics unfolds in a typical structure resembling the flower of calla lilly (shaded region in Fig. 3a). In this bistable region, the presence of noise can result in switching between the silent and the spiking state despite the constancy of the input (Fig. 3b). When the temperature is increased and spiking switches from the classical SNIC to the homoclinic regime, the occurrence of such an intermittently-interrupted firing mode is a prediction of the model analysis. With the noise stimulus appropriately chosen, see Supplementary Fig. S6, this firing mode can, therefore, also be used as a hallmark of homoclinic spike generation that distinguishes it from the classical SNIC case. For completeness, we note that inputs strong enough to trigger the excitation block (or depolarization block), thereby silencing the neurons, are not considered here.

### Experimental hallmarks of homoclinic firing with temperature increase

To explore whether a temperature increase can switch spike generation to a homoclinic regime, we performed whole-cell recordings from CA1 pyramidal neurons in mouse hippocampal slices in the absence of synaptic input at different temperatures of the bath solution and looked for the two theory-predicted indicators of homoclinic firing: (i) the intermittently-interrupted mode resulting from neuronal bistability and (ii) an increase of the locking range (resulting from the switch to an asymmetric PRC).

To assess the dynamical type, we quantified both the locking range via the PRC (as an indicator of the synchronization ability) as well as the fraction of pauses in regular firing (as an indicator of the bistability, see “Methods”). The latter captures the relative duration of particularly long ISIs, thus yielding zero or very small values for regularly firing neurons, while increasing with the number and duration of firing pauses in an intermittently-interrupted firing mode. Using a noise stimulation protocol, spiking responses were measured at each of two temperatures (32 °C and 38–41 °C, respectively); cells fired regularly at the lower temperature. The voltage trace of a simulated and an experimentally measured cell at both temperatures can be compared in Fig. 3b, d. While firing regularly at the lower temperature, both the model and the CA1 cell show a significant fraction of interrupted firing intervals at the higher temperature. The data showed that the switch from regular to interrupted firing patterns can occur within a range as narrow as 2 °C, see Supplementary Fig. S8.

Due to the high demands on recording stability for experimental PRC measurements and the requirement of a reasonable level of intrinsic noise (see “Methods”), estimating PRCs in general poses a challenge and thus affected our ability to characterize the PRC-derived locking range. Moreover, for our protocol, we needed to quantify PRCs at two different temperatures in cells that exhibit SNIC dynamics (judged by a 1-minus-cosine shape of the PRC) at the lower temperature. Figure 3 presents the experimental data for three cells in which we succeeded to reliably estimate both quantities (locking range and fraction of pauses) under these conditions; we note that a total of 38 cells was recorded. Of the three cells, two demonstrated an increase of the locking range at the elevated temperature (Fig. 3e), one showed a decrease. The temperature-induced change in the fraction of pauses correlated with the locking-range measurements (Fig. 3f). For both measures (locking range and fraction of pauses), the quantitative changes in two cells thus were consistent with the temperature-induced switch from SNIC to homoclinic dynamics observed in model simulations (Fig. 3e, f).

Our assumption was that most neurons operate far from homoclinic spiking to avoid pathological synchronization and hence, we did not necessarily expect to observe the switch when increasing temperature only slightly above the physiological level. Surprisingly, we found examples of cells that showed both signatures of homoclinic spiking at these elevated temperatures.

### Synchronization patterns in neural networks due to a temperature-induced switch in spike-generation type

To demonstrate the impact and relevance of homoclinic spike generation on collective dynamics, we finally turned towards networks of weakly coupled model neurons with homoclinic spike generation. The characteristic asymmetry of the PRC and the associated increase in locking range, according to the theory of coupled phase oscillators^18^, leads to a facilitated time-locking of neurons in all-to-all coupled networks. For excitatory networks, the resulting network state is frustrated^12^ (i.e., showing diverse synchronization patterns with, at least transiently, fixed, yet potentially offset phase relationships across neurons), and for inhibitory networks, an in-phase synchronization across all neurons can be expected (compare also Fig. 1b).

**Fig. 4:**
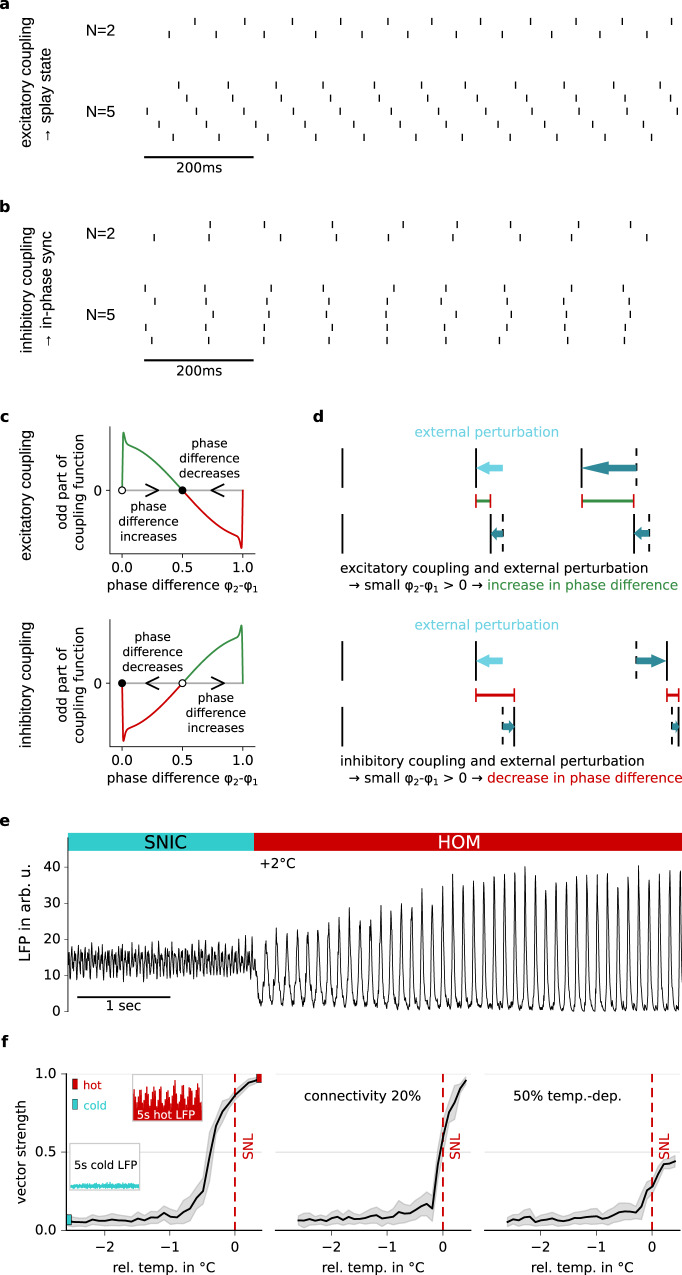
Network synchronization increases when the critical temperature is crossed. **a** Synchronization patterns for *N* = 2 or *N* = 5 model neurons with excitatory all-to-all coupling show anti-synchronization and a splay state, respectively. **b** Synchronization patterns for *N* = 2 or *N* = 5 model neurons with inhibitory all-to-all coupling show in-phase synchronization. **c** Typical shapes of the odd part of the coupling function (see “Methods”) for homoclinic neurons coupled with an excitatory pulse (top) or an inhibitory pulse (bottom). **d** Illustration of the temporal divergence of the phase difference following a small excitatory (top) or inhibitory (bottom) perturbation of the synchronized state. **e** Network simulations of 100 pulse-coupled Wang–Buzsáki neurons: the LFP population signal exhibits strong changes in synchronization in response to a temperature elevation of 2 °C (that switches spiking from SNIC to HOM). **f** Systematic effect of temperature on network synchronization: vector strength across cells as a function of time; mean +/− SD; statistics obtained across the final 40 s of the respective network simulation. Uncoupled neurons were tuned to 10 Hz firing; dashed vertical line (SNL) marks the switch between the dynamical types. Synchronization increases at the switch (left: fully coupled networks; insets: LFPs at two temperatures). This effect prevails for reduced network connectivity (middle: 20% random connectivity) and if spiking changes only in a fraction of neurons (right: fully coupled network where the temperature of only 50% of the neurons was increased).

The sign of synaptic connections (i.e., excitatory versus inhibitory) strongly influences the expected network dynamics, because the preferred phase relations between neurons in the network are different. Coupling of two homoclinic neurons with excitatory synapses leads to an alternating spiking pattern in antiphase synchronization (Fig. 4a, *N* = 2). Spikes are distributed with maximal phase distance between each other. For a low number of excitatory coupled neurons, this leads to a so-called splay state, marked, after sorting, by a staircase-like spike pattern (Fig. 4a, *N* = 5). For large networks, this pattern becomes highly sensitive to noise, as the spiking of neurons can be reordered in time by noise on the order of the phase distance between two neurons, which decreases with increasing network size. With inhibitory coupling, neurons align their spikes in time, i.e., they show in-phase synchronization independent of network size (Fig. 4b).

To strengthen the intuition, these network behaviors can be deduced from an illustration of two coupled neurons. The coupling function captures the effect of both the phase-susceptibility of the post-synaptic neuron (as measured by the PRC) and the synaptic kinetics on the phase relation of the two neurons, and is defined as a correlation between PRC and synaptic kernel. Stable phase relations are expected at phases where the odd part of the coupling function is zero and has a negative slope. In the case of two neurons with excitatory coupling, this happens at phase 0.5, for inhibitory neurons at phase 0 (Fig. 4c). In other words, two inhibitory neurons tend to fire at the same phase, whereas two excitatory neurons tend to be offset by half a cycle. Figure 4d illustrates the impact of two neurons’ respective spikes (marked as perturbation) on the timing of their subsequent spikes: For phase differences with positive (negative) values of the coupling function, the difference in firing phase is increased with each spike (decreased) compared to the preceding spike. For excitatory coupling this means that a phase difference (between temporally succeeding spikes of the two neurons) below 0.5 will further increase in the next step, whereas a phase difference above 0.5 will further decrease, resulting in a long-term phase difference of 0.5, corresponding to the synchronization pattern shown in Fig. 4a, *N* = 2 (antiphase synchronization). In contrast, for inhibitory coupling, a phase difference below 0.5 decreases, and above 0.5 increases, in the long-term resulting in a phase difference of 0 and network patterns as shown in Fig. 4b, *N* = 2 (in-phase synchronization).

Indeed, for excitatory networks with many neurons, the described dynamics lead to frustrated states. As described earlier, excitatory neurons separate their firing phases as much as possible from each other. For larger networks, however, it is impossible to identify stable phase relations allowing for large phase differences between all neurons. Consequently, states in which some neurons fire in synchrony, thus allowing others to fire at larger phase differences, can also be stable and—depending on noise levels—changes between these (anti-)synchronized groups can be induced, resulting in transient network synchronization patterns of high complexity^12^. For this reason, a pure splay state is less likely to occur with increasing network size; the continued switching between different splay state configurations may even resemble an asynchronous state (in particular for larger noise and network size). Even in the splay state of smaller networks (where a sufficient phase difference between neurons can be more easily maintained), perturbations can swap the order in which neurons fire, in particular when all neurons are identical and so is their coupling (mathematically, in this case all permutations in neuronal firing sequence constitute equivalent, equally likely network attractor states which the perturbation can navigate).

The situation is less complex for inhibitory networks. Here, homoclinic neuronal dynamics induce in-phase synchronization, with a much lower dependence on network size *N*. To demonstrate how a switch to homoclinic neuronal dynamics can affect network behavior, we explored the dynamics of a network of *N* = 100 temperature-dependent Wang–Buzsáki neurons with inhibitory coupling, estimating the local field potential (LFP) by a linearly filtered population activity (see “Methods”). The initial temperature was transiently elevated by 2 °C—an increase that was in this case sufficient to switch spike generation for the classical SNIC type to the homoclinic regime. As a consequence, network synchronization (visible in the large-amplitude LFP oscillations) was drastically enhanced (Fig. 4e). The increase evolved over several seconds; when lowering the temperature again, the synchronization slowly decreased back to its original level, see Supplementary Fig. S9.

In addition to the large-amplitude oscillations in the LFP, the in-phase synchronization of networks can be quantified by the vector strength (determined from the distribution of firing phases across all spikes, see “Methods”). A systematic analysis of vector strength as a function of temperature confirmed the increase in network synchronization toward the critical transition to homoclinic firing (Fig. 4f). Maintaining a firing rate of 10 Hz for the isolated neurons in these cases ensured that the increase in synchronization arose from more synchronized timing rather than higher firing rates. The effect prevailed for a lower degree of network connectivity (20% random connections in Fig. 4f), demonstrating its robustness. A substantial increase of the network synchronization in the vicinity of the critical temperature also emerged when only 50% of the neurons were endowed with temperature-sensitive dynamics while the other neurons remained unaffected (Fig. 4f). Overall, the network simulations demonstrate that homoclinic spikes result in a strong in-phase synchronization of inhibitory networks—an effect that becomes particularly apparent when switching the spike type. Excitatory networks with homoclinic spikers are frustrated, resulting in patterns with (transiently) fixed phase relations across neurons, such as splay states (which in the mathematical community can also be termed synchronized patterns, although in the biological community they usually are not). In the vicinity of the switch, strong changes in network synchronization patterns can be induced by relatively small changes in parameters, like a mild increase in temperature.

## Discussion

Depending on their dynamical properties, action potentials can be classified into three different types, one of which has been largely neglected in neuroscience research. In this study, we demonstrate that (i) in conductance-based model neurons the switch from the one of the classical types to this homoclinic type of spiking can be systematically induced by elevations in temperature and (ii) the switch is accompanied by systematic changes in a neuron’s temporal sensitivity to inputs (reflected in the PRC and the locking range). Our experimental measurements in hippocampal neurons shows an induction of homoclinic firing at the higher temperature for some cells, suggesting that the switch in action-potential type can be induced in mammalian neurons. Moreover, our modeling work shows that homoclinic neuronal dynamics result in network synchronization patterns, such as splayed-out states resulting from frustrated dynamics for excitatory and in-phase synchronization for inhibitory connectivity. In the vicinity of the homoclinic regime, small parameter changes can suffice to trigger strong synchronization of inhibitory networks when one of the classical spiking dynamics switches to the homoclinic type.

While PRC asymmetry can also occur in one of the two classical types, for homoclinic dynamics we encounter an unusual property: the temporal sensitivity to inputs (reflected in the PRC) is strongest directly following the spike, i.e., in a period usually considered refractory. On the one hand, this characteristic of homoclinic spiking bears consequences for encoding^23^, reflected in the spike-triggered average (STA, Fig. 1a). The STA prompts the hypothesis that homoclinic spikes can favor the transmission of high frequencies, due to the neuron’s enhanced sensitivity (non-zero PRC) directly after the spike, which allows homoclinic spikes to encode temporally close inputs in its subsequent spike timing. On the other hand, the asymmetry of the homoclinic PRC can also facilitate network synchronization. Indeed, one of the two classical types— associated with the subcritical Hopf bifurcation—can equally exhibit a PRC asymmetry and has been shown to favor synchronization. Due to the shape of the Hopf-related PRC (with spike-advancing phases occurring only later in the interspike interval), however, for this spiking type the in-phase synchronization unfolds in excitatory networks. In contrast, the specific PRC asymmetry encountered in the homoclinic regime favors in-phase synchronization in inhibitory networks and frustrated states in excitatory networks.

There is an important distinction between transitions among the two classical spike-generation mechanisms and the transition from SNIC to homoclinic spiking: the transition from SNIC to homoclinic spiking is instantaneous due to the adjacency of both regimes (separated by a codimension-2 bifurcation, see “Methods”), while the former transition requires a sequence of intermediate bifurcations to traverse from the SNIC onset to the subcritical Hopf bifurcation. Here, we show that temperature can ubiquitously transform SNIC spikers into homoclinic spikers, based on fundamental biophysical properties common to all neurons, despite differences in the quantitative temperature responses of different neuron types^24^. The transition from SNIC spikers to homoclinic spikers with increasing temperature is primarily due to the acceleration of channel gating, leading to a decreased timescale separation of the dynamical variables. Any parameter that either speeds up ion-channel gating (like a decreased gating time constant, changes in steady-state conductances, etc.), or slows down voltage dynamics (like an increase in membrane capacitance, change in reversal potentials, etc.) results in a relative change in the timescales between fast and slow dynamics of the system, see Supplementary Fig. S1. This has direct consequences and moves neurons with SNIC dynamics closer toward the homoclinic regime. Extrapolating the effect, we can assume that there are many more physiological parameters that lead to homoclinic spiking, see Supplementary Figs. S1–S3. Among these, membrane capacitance and membrane leak are most obvious^19^. As a consequence of the “direct neighborhood” of the classical SNIC and the homoclinic firing regimes in parameter space, and the associated qualitative change in PRC asymmetry, in this vicinity small parameter changes are expected to result in strong changes in network synchronization patterns. In contrast, switches between the classical SNIC and subcritical Hopf dynamics require comparatively larger parameter changes. Drastic changes in synchronization appear before the subcritical Hopf bifurcation occurs at the Bogdanov–Takens point, since the stable limit cycle is not changed at this point. Before and after the Bogdanov–Takens point, the stable limit cycle arises from a fold-of-limit cycle bifurcation, and only the fixed-point qualitatively changes its properties. Therefore, effects on synchronization (in this case in excitatory networks) are expected to be more gradual in nature. For completeness, starting from a SNIC bifurcation, also an increase in the relative timescale between voltage and gating dynamics can result in homoclinic spiking (sometimes called big homoclinic loop).

We experimentally searched for features of homoclinic firing in hippocampal pyramidal cells because pyramidal neurons, unlike hippocampal interneurons, show relatively low levels of noise and at least a subset exhibits SNIC dynamics at non-elevated temperatures, a prerequisite for our analysis. We note that, mathematically also, neurons with subcritical Hopf dynamics exhibit a (usually narrow) bistability at the threshold. The potentially resulting burst-like firing behavior, however, displays multimodal ISI distributions as well as subthreshold oscillations^25^, both of which were not obvious in the experimental data. Moreover, starting out with SNIC dynamics at the colder temperature, an immediate change to the subcritical Hopf would mathematically not be expected (see arguments about the subcritical Hopf above). Whether interneurons also exhibit homoclinic firing with its interesting coding properties, or at least SNIC firing at the colder temperature (such that the transition to homoclinic firing could be expected with an increase in temperature), is an open question for future research. Our modeling work predicts that homoclinic spiking can be universally obtained in a broad range of cells.

The ability to switch the spiking type to the homoclinic regime assigns a causal and strongly amplifying role to microscopic cellular properties on macroscopic network synchronization. While our results suggest that the described effects may extend well beyond all-to-all coupled network regimes, the effect of homoclinic spikes on macroscopic activity in networks including excitatory coupled neurons or more complex topologies merits further investigation, for example regarding complex behaviors such as chimera states^26^. Homoclinic spiking thus offers new perspectives on network synchronization in different physiological and pathological triggering situations, including neuromodulation and seizure onset^27,28^. Therefore, homoclinic spiking could provide a unifying mechanistic framework for the previously unexplained strong effects of minimal changes in biophysical parameters on network synchronization^29–32^, including a rise in temperature increase during febrile seizures^33–35^. Inhibitory neuron activity, in particular, was recently found to directly precede seizure onset^27^, strengthening hypotheses that assign inhibition a causal role in triggering synchronization. Taken together, our results suggest a functional relevance and, due to its generic inducibility, a previously underestimated prevalence of homoclinic spiking in the brain, which calls for a further exploration of this interesting activity regime.

## Methods

### Conductance-based model neurons with temperature dependence

Conductance-based model neurons describe the dynamics of the membrane voltage *v* by a current-balance equation of input current, capacitive current, and ionic current. The ionic current, ${I}_{{{{\rm{ion}}}}}={I}_{{{{\rm{ion}}}}}(v,{m}_{i},...)$, depends on *v* and the open probability of ion channels given by their gating variables, *m*_*i*_. The gating is typically modeled by first-order kinetics. The complete model is given by

1 $$\left(\begin{array}{c}\dot{v}\\ {\dot{m}}_{i}\\ ...\end{array}\right)=\left(\begin{array}{c}\frac{1}{{C}_{{{{\rm{m}}}}}}({I}_{{{{\rm{in}}}}}-{I}_{{{{\rm{ion}}}}}(v,{m}_{i},...))\\ \frac{{m}_{i}^{{{{\rm{\infty }}}}}(v)-{m}_{i}}{{\tau }_{{m}_{i}}(v)}\\ ...\end{array}\right),$$

with membrane capacitance *C*_m_ and input current *I*_in_, and where the dot · denotes the derivative with respect to time. We used three different conductance-based neuron models to illustrate the generality of our results. As a two-dimensional model for Fig. 2e, the Izhikevich sodium–potassium model with voltage dynamics and a single gating variable for potassium was chosen^5^. As a three-dimensional model, a variant of the Wang–Buzsáki model with gating variables for potassium and sodium inactivation was selected, which was originally designed for hippocampal interneurons^36^. The four-dimensional model is a variant of the Traub–Miles model with an additional gating variable for sodium activation, which was originally fitted to hippocampal pyramidal cells^37^. In Fig. 4, we used the variant of the Wang–Buzsáki model.

Temperature affected the models in three ways: via ionic reversal potentials, peak conductances, and gating rates. We introduce the same dependence on temperature in each of the models. With a temperature increase of 10 °C, the gating rates $1/{\tau }_{{m}_{i}}$ were increased by a factor four (Q_10_ = 4), the maximal conductances *g*_max_ were increased by a factor 1.3 (Q_10_ = 1.3), and the reversal potentials were shifted according to the Nernst equation. Thus, the models captured the major changes observed experimentally for temperature alterations. For simplicity, we assumed that the scaling of the parameters is equal for all gating variables. The effect of temperature on neurons obeys typical biophysical laws, which are thought to underlie all neurons^38^. Arrhenius’ scaling laws describe the influence of temperature on ion-channel kinetics, while the temperature dependence of aquatic diffusion sets the effect on channel conductances^38^. While these laws are universal, the exact Q_10_ value which sets the scale of change per 10 °C may vary between neurons. Still, the universality of the Arrhenius law and aquatic diffusion lead us to expect that the temperature effects modeled here are valid for a wide class of neurons.

The transition from SNIC to homoclinic spiking happens at the codimension-two saddle-node-loop (SNL) bifurcation^19^. Both SNIC and homoclinic spike classes show a saddle-node bifurcation with an increase in input current (blue line in Fig. 3a), which eliminates the resting state. The elimination of the resting state coincides with spike initiation at a SNIC bifurcation (Fig. 2a, left), while homoclinic spiking is initiated at lower inputs (Fig. 2a, right), leading to bistability between rest (Fig. 2a, right panel, filled dot) and spiking (Fig. 2a, right panel, red trace).

The critical temperature of interest, at which the switch from SNIC to homoclinic spiking occurs, depends on the model parameters. To compare the increase in synchronization ability (i.e., locking range, see below) for all three models, we aligned the model temperatures to the SNL temperature and investigated the region below that spans about three degrees Celsius. The temperature of 0 °C in this scale, at which the SNL bifurcation occurs, corresponded for the Izhikevich sodium–potassium model to a temperature difference (above the original model) of Δ*T* = 16.95 °C, for the Wang–Buzsáki model to Δ*T* = 3.59 °C, and for the Traub–Miles model to Δ*T* = 31.14 °C. These critical temperatures were identified by visual inspection of numerical voltage traces (continuation software AUTO)^39^; given the low dimensionality of the models, the SNL bifurcation removed the afterhyperpolarization.

### Phase responses

The phase reduction allows to simplify conductance-based model neurons to one dimension with a single variable, the phase *φ*^18^. The phase increases from zero to one between the voltage maxima of two consecutive spikes for the unperturbed neuron, proportionally to the elapsed time. A neuron perturbed by a small input responds with a phase shift, i.e., a delay or advance of the next spike. The phase-response curve (PRC) relates the phase, at which the input occurs, to the phase shift of the subsequent spike. The PRC is also known by the names phase resetting curve, phase sensitivity, or phase susceptibility. The first-order phase dynamics, subject to a time-dependent, weak input stimulus, *s*(*t*), is given by

2 $$\dot{\varphi }=f+Z(\varphi )s(t),$$

with baseline firing rate, *f*, and PRC, *Z*(*φ*).

For the unperturbed system in Eq. (1), i.e., with constant input *I*_in_, the PRC is given by the voltage component of the solution to the associated adjoint system^4,18^, which can be determined from Eq. (1). The adjoint equation is given as $\dot{Z}={J}^{{{{\rm{\top }}}}}(x(t))Z$, with *J* the Jacobian of the original system, evaluated along the limit cycle trajectory given by *x*(*t*). We solved the adjoint equation with appropriate boundary conditions (periodicity in PRC and limit cycle) using the continuation software AUTO^39^. The phase-response curve is the voltage component of the solution *Z*(*t*) to the adjoint equation, as reviewed in ref. ^4^. We rescaled time *t* to phase *φ* by dividing the time by the limit cycle period. To comply with Eq. (2), the PRC *Z*(*φ*) was normalized as $Z(\varphi )\cdot F(\varphi )=f,\forall \varphi$.

### Synchronization measure based on single-cell characteristics

Synchronization between neurons depends on their ability to influence each other. Based on the theory of weakly coupled oscillators, the PRC allows to predict the synchronization behavior of a network of coupled model neurons^18^. The relation between PRC and synchronization can be illustrated for two oscillators with firing rates *f*_*i*_ and *f*_*j*_. For a given (synaptic) coupling kernel, *G*(*t*), the dynamics of the phase difference between both oscillators, $\psi ={\varphi }_{i}-{\varphi }_{j}$, is given as

3 $$\dot{\psi }=\varDelta f+H(\psi )-H(-\psi ),$$

with $\varDelta f={f}_{i}-{f}_{j}$, where the coupling function $H(\psi )={\int }_{0}^{1}Z(\varphi )G(\psi -\varphi ){{{\rm{d}}}}\varphi$ results from an averaging over the phase in the time frame of slow phase differences^18^. The locking range (or entrainment range), ${{\max }}_{\psi }(H(\psi )-H(-\psi ))-{{\min }}_{\psi }(H(\psi )-H(-\psi ))$, gives the maximal phase detuning Δ*f* for which Eq. (3) has a stable fixed point, $\dot{\psi }=0$, corresponding to phase synchronization.

Theoretical considerations show that the locking range translates to phase synchronization in weakly coupled Kuramoto networks^18^. The larger the locking range of individual model neurons, the more easily they are expected to synchronize when coupled in a network. The locking range also specifies the frequency range across which a neuron can reliably couple to a periodic input.

The locking range in Fig. 2e refers to model neurons with an infinitesimal short coupling, $G(t)=A\delta (t-{t}_{{pre}})$, with presynaptic spikes at *t*_*pre*_ and amplitude *A* = 5 pA/cm^2^. In this case, $H(\psi )={AZ}(\psi )$, and the min–max difference is taken over the odd part of the PRC, i.e., over $Z(\psi )-Z(-\psi )$. Consequently, the locking range increases with the asymmetry of the PRC and enlarged PRC asymmetry underlies the increase in locking range around the SNL bifurcation^19^. For completeness, we note that, mathematically, the odd part is defined as half of ($Z(\psi )-Z(-\psi )$); we here neglect the factor 0.5 for clarity of the argumentation and without effect on the results presented. The increase of the locking range when switching to the homoclinic regime also holds for synaptic coupling at physiologically realistic timescales, see Supplementary Fig. S4.

### Synchronization in network simulations

Figure 4 shows that synchronization is enhanced in networks of homoclinic neurons^19,40,41^. Network dynamics were simulated using the simulation environment *brian2*^42^. A number of *N* temperature-dependent inhibitory Wang–Buzsáki neurons were stimulated with a mean current of *I*_in_ and a zero-mean white-noise current with a standard deviation of $\sigma =12\,{{{{\rm{pA}}}}/{{{\rm{cm}}}}}^{2}\sqrt{{{{\rm{ms}}}}}$. The neurons were coupled with voltage perturbations of amplitude $\epsilon$. The parameters for Fig. 2d are *N* = 10, *I*_in_ was adapted for each temperature to ensure spiking at around 10 Hz, *σ* adapted without coupling to a coefficient of variation of *CV* = 0.08, and $\epsilon =-44\,\upmu {{{\rm{V}}}}$, temperature of the red traces was 3.5 °C warmer than for the blue. For Fig. 4e*N* = 100, ${I}_{{{{\rm{in}}}}}=300\,{{{{\rm{pA}}}}/{{{\rm{cm}}}}}^{2}$, $\sigma =12\,{{{{\rm{pA}}}}/{{{\rm{cm}}}}}^{2}\sqrt{{{{\rm{ms}}}}}$, and $\epsilon =-2\,\upmu {{{\rm{V}}}}$, with temperatures above the original Wang–Buzsáki model of *Δ*T = 2 °C and *Δ*T = 4 °C, which corresponded to relative temperatures of −1.59 °C and 0.41 °C with respect to the model’s transition temperature from SNIC to homoclinic firing; for Fig. 4f*N* = 100, *I*_in_ was adapted for each temperature to ensure spiking at around 10 Hz, *σ* adapted without coupling to a coefficient of variation of *CV* = 0.02, and $\epsilon =-2\,\upmu {{{\rm{V}}}}$. The network simulations were started with random initial voltages and run for 5 s with a high noise input (five times *σ*) to desynchronize the neurons. After another 5 s of simulation with a normal noise level, the neurons were coupled and the actual simulation started (length ≈100 s). Figure 4e and the insets in Fig. 4f show a proxy for the LFP based on the simulated spike pattern. Each spike in the network added an exponential decay with 10 ms time constant to the local field potential, ${{{\rm{LFP}}}}=\mathop{\sum}\limits_{i}{\exp }(-(t(t > {t}_{i})-{t}_{i})/(10\,{{\mbox{ms}}}))$, where *t*_*i*_ denotes the spike times for all neurons of the network. The synchronization of the network was measured by comparing the phases *φ*_*j*_ of the individual model neurons: The vector strength, a common measure of synchronization of rhythmic neurons, $\mathop{\sum}\limits_{j}{\exp }(2\pi i{\varphi }_{j})$, increases with enhanced in-phase synchronization, and is also known as order parameter^18^. The vector strength across all neurons in the network was evaluated at each point in time based on a linear phase interpolation between subsequent spikes. Temporal mean+/− SD were obtained from the final 40 s of each simulation.

### Experimental procedures

#### Ethics statement

Animal maintenance and experiments followed institutional guidelines, the guidelines of the Berlin state (T0100/03), and European Union (EU) Council Directive 2010/63/EU on the protection of animals used for experimental and other scientific purposes. Mice were housed in a laboratory animal facility at an ambient temperature of 18–23 °C and 45–55% humidity. A 12-light/12-dark cycle was used (light cycle 6 AM to 6 PM).

#### Slice preparation and storage

C57BL/6N male mice of age between 10 and 13 days and between 22 and 30 days were decapitated following isoflurane anesthesia. Brains were transferred to ice-cold sucrose-based ACSF containing the following (in mM): 87 NaCl, 2.5 KCl, 3 MgCl_2_·6H_2_O, 0.5 CaCl_2_, 10 glucose, 50 sucrose, 1.25 NaH_2_PO_4_, and 26 NaHCO_3_ (pH 7.4). Horizontal slices (400-μm thick) of ventral to mid-hippocampus were cut using a vibratome (VT1200S, Leica) and stored in an interface chamber perfused with standard ACSF containing the following (in mM): 119 NaCl, 2.5 KCl, 1.3 MgCl_2_, 2.5 CaCl_2_, 10 glucose, 1.25 NaH_2_PO_4_, and 26 NaHCO_3_, at pH 7.4 and an osmolarity of 290 to 310 mosmol/l. The temperature was kept at 32 to 34 °C, and the slices were superfused with a flow rate of about 1 ml/min. ACSF solutions were equilibrated with carbogen (95% O_2_, 5% CO_2_). Slices were allowed to recover for at least 1.5 h after preparation.

#### Electrophysiology

For recordings, we mounted the slices on polylysine-coated glass coverslips and transferred them to the recording chamber, which was perfused at a rate of 5–6 ml/min. The recording chamber was placed in an Olympus BX-51WI upright microscope, and slices/cells were visualized at ×4 and ×60, respectively^43^. Whole-cell recordings were performed with glass electrodes (2–5 M*Ω*) filled with the following intracellular solution containing (in mM): 120 K-gluconate, 10 HEPES, 10 KCl, 5 EGTA, 2 MgSO_4_·7H_2_O, 3 MgATP, 1 Na_2_GTP, 14 phosphocreatine, and 5.4 biocytin (0.2%). The pH was adjusted to 7.4 with KOH. Synaptic activity was blocked with the GABA_A_ receptor antagonist SR-95531 (gabazine) and glutamate receptor blockers (NBQX and d-APV). The synaptic blockers turned out to be temperature-dependent; in order to block synaptic activity also at higher temperatures, the blocker concentration was set to 20 μM SCH-50911, 5 μM SR-95531 (gabazine), 25 μM NBQX and 100 μM d-APV. Whole-cell data were amplified tenfold for current-clamp recordings using a MultiClamp 700B amplifier (Molecular Devices). Signals were low-pass filtered at 3–6 KHz using the built-in Bessel filter of the amplifier and digitized at 10 KHz with 16-bit resolution using an analog-to-digital converter (Digidata 1550 A, Molecular Devices). Data were sampled and stored using the Clampex software (version 10.7, PClamp Software Suite, Molecular Devices).

#### Spiking recorded in response to noise input

Neurons were stimulated with a step current adapted to obtain repetitive spiking with a firing rate between 5 and 10 Hz, and an additional noise current with zero mean. The noise current represented an Ornstein–Uhlenbeck process with a time constant of 4 ms, meant to simulate typical synaptic timescales. The standard deviation of the noise was adapted such that the spike train showed higher variability than without noise (visual inspection). The membrane voltage was recorded and spikes were identified based on a voltage threshold at −20 mV. Spike trains were obtained in 38 cells at low (around 32 °C) and high (mostly around 40 °C, some at around 37 °C) temperatures. For some cells, also recorded intermediate temperatures were recorded. After changing the temperature of the bath, the slice was allowed to adapt for at least 2 min before the recording started.

### Phase-response curve measurement from recorded spiking

The recorded data were analyzed using Spyke Viewer^44^ extended with additional plug-ins for PRC measurements. The deviations in spiking induced by the noise current were used to quantify the PRCs. PRCs can be estimated from the deviations in the mean firing rate resulting from the perturbations caused by the injected noise. For the experimental PRCs and derived measures shown in this article, an adaptation of the so-called STEP method based on a minimization of spike-time prediction errors was used^45^. Given a voltage trace in response to an experimentally known noise stimulus, the STEP method consists of the following steps:

1. Identification of spikes by threshold crossing at ${t}_{i},i=0,1,...,N-1$ where *N* is the number of spikes in response to the noise stimulus.
2. Calculation of interspike intervals (ISIs), ISI_*i*_ $={t}_{i+1}-{t}_{i},i=0,1,...,N-2$.
3. Calculation of the phase deviations, $\varDelta {\varphi }_{i}=({{\langle }}{{{\mbox{ISI}}}}{{\rangle }}-{{{\mbox{ISI}}}}_{i})/{{\langle }}{{{\mbox{ISI}}}}{{\rangle }}$, based on the mean ISI, ${{\langle }}{{{\mbox{ISI}}}}{{\rangle }}=1/(N-1)\mathop{\sum}\limits_{i}{{{\mbox{ISI}}}}_{i}$.
4. Cutting of the noise stimulus in snippets *n*_*i*_, such that each *n*_*i*_ is the noise stimulus between the spikes at *t*_*i*_ and *t*_*i*+1_, and rescaling of the time such that each noise snippet goes from phase *ϕ* = 0 to 1 (which corresponds to the time interval of one mean ISI).
5. Definition of PRC *Z* as a sum of base functions. We used trigonometric base functions, $Z(\varphi )={a}_{0}+\mathop{\sum }\nolimits_{k=1}^{5}{a}_{i}{\cos }(k2\pi \varphi )+\mathop{\sum }\nolimits_{k=1}^{5}{b}_{i}{\sin }(k2\pi \varphi )$.
6. Optimization of the PRC *Z* such that the PRC minimizes for all ISIs the prediction of the phase deviation based on the stimulus in the interspike interval, i.e., finding a PRC that optimizes $\varDelta {\varphi }_{i}={{\langle }}{{{\mbox{ISI}}}}{{\rangle }}\int Z(\varphi ){n}_{i}(\varphi ){{{\rm{d}}}}\varphi$. We used a least-squares algorithm to optimize the PRC coefficients. Based on a temporal discretization, the STEP algorithm arranges all information in a large matrix that simplifies the optimization, see ref. ^45^.

A bootstrap of the data with 200 repetitions was used to provide error estimates for the measured PRCs. We first estimated the PRC based on half of the recorded spikes, chosen randomly from the set of all spikes. The resulting error was propagated to the locking range, and used to plot the error bars shown in Fig. 3e. We then estimated the PRC based on a spike train resulting from randomly shuffled ISIs. The resulting error sets the noise level around zero above which a reliable PRC measurement should rise.

Commonly, experimental measurements of PRCs rely on short current perturbations at a one-time point per ISI. We here use a continuous noise stimulus instead, in order to prevent misinterpretation of the PRC shape: Using single time point perturbations, measured PRCs are known to be shifted to the left if the stimulus strength is too large^18^. The resulting PRCs might be mistaken for homoclinic PRCs. The noise stimuli protocol chosen here avoids this artifact and possible misinterpretation, as too strong a stimulus strength results in a right shift in the measured PRCs^46^.

In order to obtain reliable PRC estimates, neuronal spiking has to be sufficiently stationary^47,48^. As the recorded pyramidal cells showed spike adaptation within the first seconds of a current step stimulation, we used recording traces with 60 s of stimulation, but ignored spikes occurring in the first 5 s. While canonical PRCs are only expected for low firing rates, firing rates that are too low limit the overall number of spikes that can be recorded. We thus restricted the analysis to recordings with a mean firing rate between 3.5 and 13.5 Hz.

For three cells, we found PRCs above the bootstrap-derived noise level whose shape resembled a 1-minus-cosine as expected for SNIC dynamics^4^ at the low recording temperature around 32 °C. For these cells, also the PRCs at higher temperatures were above the bootstrap-derived noise level. The recordings used to estimate the PRC were selected based on the lowest mean firing rate *r*_cold_ of all recordings at around 32 °C. Recordings with a mean firing rate within the range ${r}_{{{{\rm{cold}}}}}\pm 0.51\,{r}_{{{{\rm{cold}}}}}$ were included in the PRC-estimation set. Recordings from this set recorded at around 32 °C were used to estimate the PRC at the low temperature (32.3 °C for cell 1, 31.9 °C for cell 2, and 32.2 °C for cell 3). The highest available temperature was used to estimate the PRC at the high temperature (37.7 °C for cell 1, 40.0 °C for cell 2, and 40.0 °C for cell 3). From the estimated PRC *Z*, which depends on the phase *φ*, the locking range was predicted based on the odd part of the PRC; the locking range is measured as ${{\max }}_{\varphi }(Z(\varphi )-Z(-\varphi ))$ under the assumption of pulse coupling (Fig. 2e).

### Evaluation of bistability from recorded spiking

Homoclinic neuronal dynamics are marked by bistability between resting state and stable limit cycle (i.e., spiking)^25^. In recordings with an appropriate amount of noise, this bistability shows as a stochastic switching between rest and spiking; elongated periods close to rest are reflected in exceptionally long interspike intervals (ISIs), i.e., firing pauses. To test whether the ISI distributions differed between high and low temperatures for the three cells identified based on the PRC measurement, the occurrence of particularly long ISIs was analyzed, based on the same recording traces as for the PRC estimation.

Voltage traces were cut into snippets of 27 s length (allowing us to obtain two snippets per recording from the 60 s with the first 5 s removed). Bistability was evaluated for a given cell and temperature by calculation of the fraction of pauses, defined as the relative duration of exceptionally long ISIs. Specifically, for each snippet the sum of all ISIs with a duration of more than twice the average ISI duration was obtained and divided by the sum of all ISIs in the snippet. To evaluate whether the fraction of pauses differed significantly between the ISI sets recorded at low and high temperatures, we used a rank-sum test (Matlab implementation rank-sum with standard parameters; two-sided Mann–Whitney *U* test). One star in Fig. 3f marks *P* values below 0.05, two stars mark *P* values below 0.01. As an alternative measure expected to increase with the occurrence of bistability, Supplementary Fig. S5 shows the coefficient of variation (CV) for the same recordings presented in Fig. 3f.

### Computational resources

For numerical simulations, data analysis, and plotting, we used the python packages scipy^49^, sympy^50^, and matplotlib^51^ (python version 2.7.16) and for numerical simulation the simulation environment *brian2*^42^ (version 2.3.0.2). In addition, we used for data analysis the tool spykeviewer^44^ (version 0.4.1), for statistical tests Matlab (Mathworks, version 9.7.0.1190202), for numerical continuation AUTO-07P (version 0.7), and for graphic design the open-source software inkscape^52^ (version 1.1).

### Reporting summary

Further information on research design is available in the Nature Research Reporting Summary linked to this article.

## Supplementary information

Supplementary Information

Reporting Summary

## Data Availability

The experimental data generated in this study have been deposited at zenodo.org under 10.5281/zenodo.6589677.

## Code availability

The programming code is textbook standard or previously published. It can be made available upon request.
