# Supplementary material for: Temperature elevations can induce switches to homoclinic action potentials that alter neural encoding and synchronization
Source: Nat Commun. 2022 Jul 8;13:3934. doi: 10.1038/s41467-022-31195-6 (PMC9270341; doi:10.1038/s41467-022-31195-6)
Supplement: Supplementary file 1 — Supplementary Information [file 41467_2022_31195_MOESM1_ESM.pdf]

SUPPLEMENTARY INFORMATION

Temperature elevations can induce switches to homoclinic action potentials that alter neural encoding and synchronization

Janina Hesse, Jan-Hendrik Schleimer, Nikolaus Maier, Dietmar Schmitz, Susanne Schreiber

1. Mathematical background on spike onset bifurcations

Neuron models commonly show two distinct dynamical states:

1. The so-called *resting state*, in which the membrane voltage remains at the same value, typically around -65 mV. Dynamically, this state corresponds to a stable fixed point, a stable node (type I dynamics) or focus (type II dynamics).

2. A state marked by continuous *spiking*, in which the membrane voltage deviates repetitively to above 0 mV. Dynamically, this state corresponds to a stable limit cycle.

The transition from rest to spiking entails two dynamical transitions, so-called bifurcations: The limit cycle has to be created (or stabilized), and the fixed point has to lose stability. For the saddle-node on invariant cycle (SNIC) bifurcation, both events happen at the same parameter value (e.g., temperature): where the saddle collides with the node in the saddle-node bifurcation, the heteroclinic loop previously connecting saddle and node becomes a homoclinic loop that connects the saddle-node to itself, and the homoclinic loop detaches as limit cycle. For the saddle homoclinic orbit (HOM) bifurcation, the limit cycle results from a homoclinic loop that connects the saddle to itself, for parameters where the stable fixed point still (co)-exists. This results in the bistability observed at the onset of homoclinic firing. For both SNIC and HOM spike onset, the homoclinic loops attached to a fixed point result in arbitrary low firing rates at spike onset, because the proximity of the fixed point enforces slow dynamics. Consequently, both SNIC and HOM dynamics have been associated with so-called type I firing, i.e., they display a frequency-input (f-I) curve that continuously increases from 0 Hz. For the HOM case (marked by hysteresis), however, this continuity only prevails when the f-I curve is measured by a gradual decrease in input. In contrast, the f-I curve in the HOM case is discontinuous when the firing threshold is crossed from below via a gradual increase in input. For a more detailed introduction into neuronal bifurcations please refer to.[1]

The transition from SNIC to HOM dynamics happens at the so-called saddle-node loop (SNL) bifurcation, also known as saddle-node homoclinic orbit, saddle-node noncentral homoclinic, saddle-node separatrix-loop bifurcation, or orbit flip bifurcation, see.[2] The transition occurs as follows, compare Fig. 2a of the main text. At the saddle-node bifurcation, the dimension along which saddle and node collide (horizontal arrow with a single arrowhead in Fig. 2a of the main text) entails considerably slower dynamics compared to the other dimension(s), and is called the semi-stable manifold. Most dynamics in the vicinity of the semi-stable manifold relax onto it before approaching the saddle node. Exceptions to this rule only occur for dynamics that approach the saddle-node via the other manifolds, accordingly called weakly-stable manifolds. These are exemplified in Fig. 2a of the main text by the vertical arrow with a double arrowhead. The double arrowhead is chosen because the dynamics along these manifolds

is considerably faster than the (infinitely slow) dynamics along the semi-stable manifold. The spike upstroke always follows the semi-stable manifold, which enforces slow dynamics at the spike upstroke (the right branch of the horizontal arrow in Fig. 2a of the main text), and thus low firing rates at spike onset. The spike downstroke can follow either of the manifolds: At the SNIC bifurcation, the homoclinic orbit connecting the saddle-node to itself returns via the semi-stable manifold; in biological terms, spike upstroke and spike dowstroke plus afterhyperpolarization happen on the same time scale, both last for the same time. At the SNL bifurcation, the approach of the saddle-node switches to the weakly-stable manifold (denoted as switch in the middle panel of Fig. 2a of the main text). In biological terms, spike dowstroke plus afterhyperpolarization occurs much faster than the spike upstroke. In fact, at spike onset the difference is arbitrarily large, such that the dynamics of the spike downstroke plus afterpolarization can be safely ignored. As a result, the interspike interval halves at the SNL bifurcation (because only the upstroke takes time), and interestingly, also the PRC is halved.[2] This effective halving of a formerly symmetric PRC at the SNIC bifurcation leads to a strong asymmetric component at the SNL bifurcation, and thus a large increase in locking range.

Going past the SNL bifurcation into the HOM regime, the homoclinic loop from which the limit cycle detaches forms already at the saddle before it collides with the stable node. Yet, even in proximity of the saddle-node in parameter space, the dynamics around the saddle look similar to those around the saddle-node, i.e., dynamics are much slower along the spike upstroke compared to the rest of the spike. Moreover, the dynamics along the upstroke resemble those along the semi-stable manifold of the saddle-node, just that the initial part, i.e., half the distance between node and saddle, has to be ignored. As a result, the initial, slowest part of the spike upstroke at the SNL bifurcation is removed at a HOM bifurcation, thus the interspike interval is further reduced, and also the PRC shape is effectively restricted to later phases of the PRC at the SNL bifurcation. This can be seen in Fig. 2b; scaling the HOM and SNIC PRC to the same amplitude, the HOM PRC has the same shape as the later part of the second half of the SNIC PRC. The second half of the SNIC PRC corresponds to the PRC shape at the SNL bifurcation. The SNL bifurcation induces a spontaneous symmetry breaking of the PRC by a sudden replacement of slow dynamics (without a linear term due to the zero Eigenvalue of the saddle-node) by fast dynamics (with a linear term).

2. Switching from SNIC to HOM firing with neuronal parameters

Starting from the SNIC regime, the HOM regime can be approached by neuronal parameters that change the relative time scale of voltage dynamics and ion channel gating. This differential speed-up distorts the periodic orbit underlying the spike. To induce the transition to the HOM regime, the limit cycle trajectory has to switch the approach of the saddle-node from the semi-stable manifold to the weakly-stable manifold. For this to happen, the spike downstroke has to pass closer and closer in parallel to the weakly-stable manifold, until the dynamics jump on the weakly-stable manifold, compare Fig. S1a. Intuitively, this approach of the weakly-stable manifold happens when the flow field along the spike downstroke, as defined by Eq. (1) in the main text, becomes steeper. This is exemplified in Fig. S1 by the location marked by the star. For the flow field along the spike downstroke to become steeper, two basic options exist:

1. The speed of potassium gating, $\dot{n}$, is increased, see Fig. S1b.

2. The speed of voltage dynamics, $\dot{v}$, is decreased, see Fig. S1c.

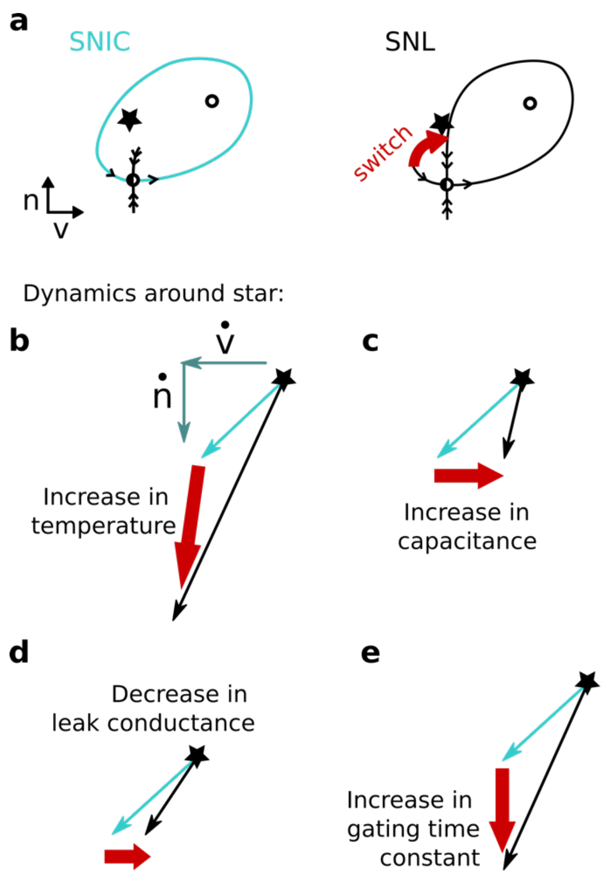

Fig. S1: Parameters can induce the transition between saddle-node on invariant cycle (SNIC) and saddle-homoclinic orbit (HOM) dynamics at the saddle-node-loop (SNL) bifurcation by effectively increasing the rate of ion channel gating compared to the voltage dynamics in a twodimensional example. **a** The transition of the approach of the saddle node from the semi-stable manifold to the weakly-stable manifold occurs at the SNL bifurcation. For this transition to happen, the flow of the dynamics has to become steeper around the area marked by a star. **b** Steeper dynamics around the star can result in the current study from an increase in temperature, which scales the ion channel gating more strongly than the conductances, thus effectively increasing $\dot{n}$ relative to $\dot{v}$. **c** An elevation of the capacitance increases the steepness of the flow by slowing the voltage speed $\dot{v}$, without altering the gating. **d** A decrease in the leak conductance increases the steepness of the flow by slowing the voltage speed $\dot{v}$. In addition, changes in leak can shift the location of the fixed points and thus the trajectory in the phase plane (not shown). **e** An increase in the gating time constant directly increases $\dot{n}$ and thus the steepness of the flow.

The voltage dynamics can be directly slowed down by an increase in capacitance, see Fig. S1c. Setting $\dot{v} = 0$ in Eq. (1) of the main text to calculate the fixed points, the capacitance can be removed from the equation, including the fixed points, and thus the saddle-node, are independent of the capacitance. This implies that the net effect on the voltage is indeed a steepening of the spike downstroke close to the saddle-node, allowing to approach the HOM regime with an increase in capacitance. This can also be seen at the membrane time constant, which is given by

$$\tau_m = \frac{C_m}{g_L}. \quad \text{(S1)}$$

The situation is more involved for the leak conductance, because the leak conductance also affects the location of the fixed points. While for the same location in state space, an increase of the leak conductance effectively increases the speed of the voltage dynamics, see Eq. (S1), the change in the position of the saddle-node fixed point effectively results in a steepening of the flow field along the spike downstroke. The ability of the leak conductance to induce the SNL bifurcation results from the mathematical analysis in.[3] The potassium gating can be accelerated without changing the location of the fixed points by an increase in the ion channel gating time constants, compare Eq. (1) in the main text and Fig. S1e. For temperature, the predominant effect is also on the gating time constant, because the gating Q_{10} value with a value of 4 are larger than the Q_{10} value of the conductances with a value of 1.3. Hence, also for an increase in temperature, the major effect is an acceleration of the gating compared to the voltage dynamics, see Fig. S1b.

All neuronal parameters that influence the relative timescales between gating and voltage, and potentially the location of the fixed points, are candidates for inducing the transition between SNIC and HOM regime. An example is provided by ion concentrations,[4] membrane capacitance[2] or the leak conductance, see Fig. S2. More generally, a change in the relative time-scale of voltage and potassium gating leads to homoclinic firing when starting from a SNIC neurons. This has been proven mathematically for two-dimensional conductance-based models, such as the sodium-potassium model used in Fig. 2e, and for models in proximity of the Bogdanov-Takens-Cusp bifurcation.[2,3]

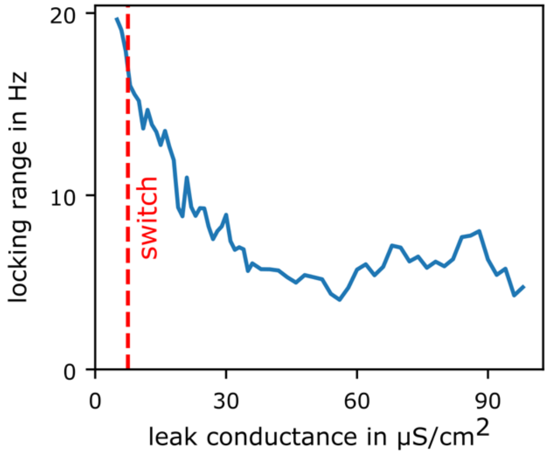

Fig. S2: A reduction in leak conductance can induce the transition between saddle-node on invariant cycle (SNIC) and saddle-homoclinic orbit (HOM) dynamics in the Wang-Buzsáki model. The transition occurs at a leak conductance of $g_L \approx 7.5$ μScm[2]. Accordingly, the locking range increases when approaching the transition from above (higher values of leak).

Any parameter that can induce the switch between SNIC and homoclinic spiking can also be used to alter the exact occurrence of the switch when varying another parameter to induce the transition. This might help to understand seemingly unconnected effects of physiological parameters on network synchronization patterns. For example, the respective contribution of increased body temperature and increased blood pH levels to febrile seizures is still an open research question.[5,6] Motivated by the correlation of febrile seizures with increased blood pH level,[7,8] we tested in a model neuron whether

alkalosis can decrease the temperature at which the transition from SNIC to homoclinic spiking occurs. The temperature-dependent Wang-Buzsáki model was supplemented with a pH dependence of the sodium ion channel. An increase of the pH level by one unit was implemented as a shift of the sodium activation curve by −3.2 mV, a shift of the sodium inactivation curve by −2.6 mV, and a 15% increase in sodium maximal conductance, based on literature values.[9,10] We find that the critical temperature, at which the transition from SNIC to homoclinic spiking occurs, is lowered by increasing pH levels, see Fig. S3. In this case, altered synchronization patterns would be expected to occur at a lower temperature when body temperature increases due to a fever. Assuming that febrile seizures could be triggered by a transition from SNIC to homoclinic spiking, this hints at the possibility that pH levels and body temperature might be two sides of the same coin for the induction of febrile seizures: Both increased pH level as well as increased body temperature shift neuronal dynamics towards homoclinic firing. The consideration of spike onset dynamics in addition to basic electrophysiological measurements such as resting membrane voltage and membrane ti me constant might in the future allow electrophysiological experimentalists to better understand the impact of physiological parameters on neuronal dynamics.

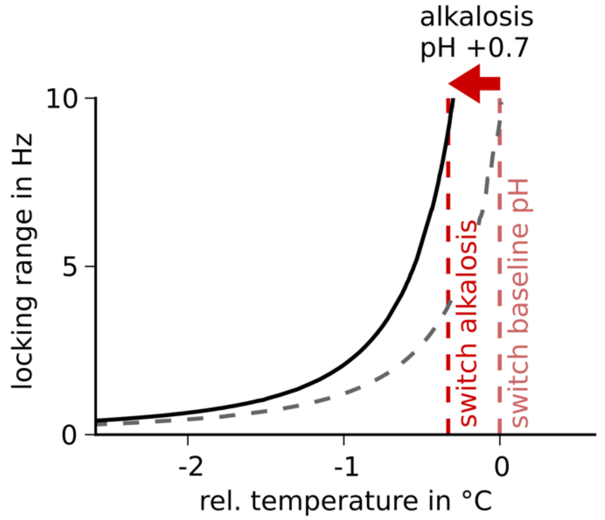

Fig. S3: Physiological parameters can change the location of the switch between SNIC and homoclinic spiking. The model shows a decreased temperature for the transition between SNIC and homoclinic spiking with a pH level increased by 0.7 units. The level of extracellullar pH affects sodium ion channels, with a shift in the sodium activation and inactivation curve to lower voltages (per pH unit 3.2 mV and 2.6 mV lower, respectively) and a change in maximal sodium conductance (increasing by 15% per pH unit).

3. Dependence of the locking range on the timescale of synaptic transmission

In order to check how the timescale of synaptic transmission affects the synchronization ability, we derived the locking range for a Wang-Buszáki neuron with a more realistic, temperature-dependent synaptic coupling (Fig. S4). For this purpose, post-synaptic currents were assumed to have an instantaneous onset followed by an exponential decay, $G(t) = I_{\mathrm{syn}}\mathrm{exp}(\frac{t-t_i}{\tau})$, with time constants $\tau =$ 6 ms for the fast, and $\tau = 50$ ms for the slow synaptic current. For illustrative purposes, we here consider inhibitory synaptic currents. The resulting inhibitory post-synaptic potentials (IPSPs) in the model were meant to mimic $\mathrm{GABA_A}$- and $\mathrm{GABA_B}$-like coupling and are shown in the inset of Fig. S4. Temperature was assumed to scale synaptic time constants τ with a factor of two for an increase in temperature of

10 °C (Q_{10}=2), as suggested by experimental observations.[11–13] Experimental studies rarely report the effect of temperature on the amplitude of the synaptic current (but see[11]). We ensured that the total charge transfer per IPSP remained independent of temperature by changing the maximal amplitude of the synaptic current I_{syn} by a factor of two for a temperature increase of 10 °C. The synaptic amplitude I_{syn} was chosen such that a single IPSP at rest resulted in a voltage deviation of ≈1mV at the SNL temperature (I_{syn} = -330 pA/cm^2 for the fast synapse and I_{syn} = -140 pA/cm^2 for the slow synapse). While a stronger increase in synaptic time constant, i.e., a pronounced slowing of synaptic transmission, tended to reduce the boosting effect on the locking range when entering the homoclinic firing regime via a temperature elevation, for synaptic transmission on the timescale of $GABA_A$ transmission a strong boost of the locking range clearly prevailed (Fig. S4).

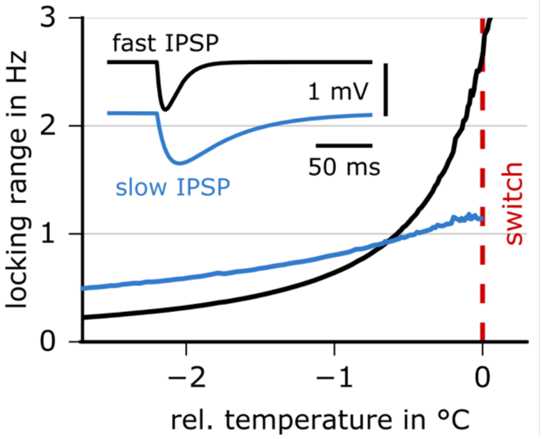

Fig. S4: Locking range assuming a postsynaptic potential with a time constant of τ = 6 ms (black) or τ = 50 ms (blue). While an increase in the synaptic time constant dampens the increase of the locking range towards the switch, the increase is still clearly visible, in particular for the faster synaptic time constant of 6 ms. The inset illustrates the time course of the inhibitory postsynaptic potentials (IPSPs).

4. Additional experimental results

4.1 Interrupted firing correlates with the coefficient of variation

Interrupted firing in the bistable range is a hallmark of homoclinic firing. While in Fig. 3 of the main text this feature is captured by the fraction of pauses, it can also be quantified by the coefficient of variation (CV). The change in CV correlates with the fraction of pauses for the mathematical model as well as the experimental cells Fig. S5.

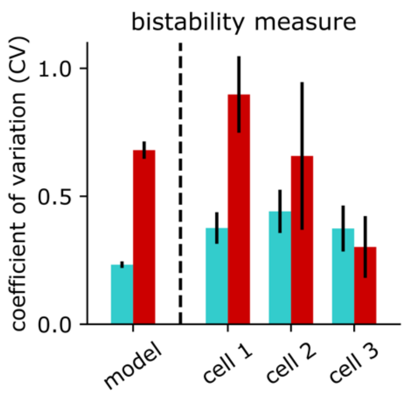

Fig. S5: The homoclinic hallmark of bistable firing also correlates with temperature-induced changes of the CV. In both the model and the cells with suspected homoclinic firing (cell 1 and cell 2) the CV (presented as mean +/- SD) is increased at the elevated temperature. In contrast, the CV does not increase in cell 3, for which also no other features of homoclinic firing could be observed. Cells are identical to those presented in Fig. 3 of the main text.

4.2 Dependence of interrupted firing on noise

We report as hallmark of homoclinic firing an increase in extremely long interspike intervals, compare Fig. 3 of the main text. Long interspike intervals can also result from high levels of noise. Fig. S6 shows the dependence of interrupted firing on noise strenght for SNIC and HOM neurons. For homoclinic firing, interrupted firing occurs already at low noise strength. For SNIC neurons, interrupted firing only occurs for large noise strength, noise levels so strong that the mean firing rate is increased above baseline. As we do not observe a strong increase in mean firing rate upon increasing the temperature in the experiments, we deem it unlikely that the increase in interrupted firing that we observe results from an increase in noise.

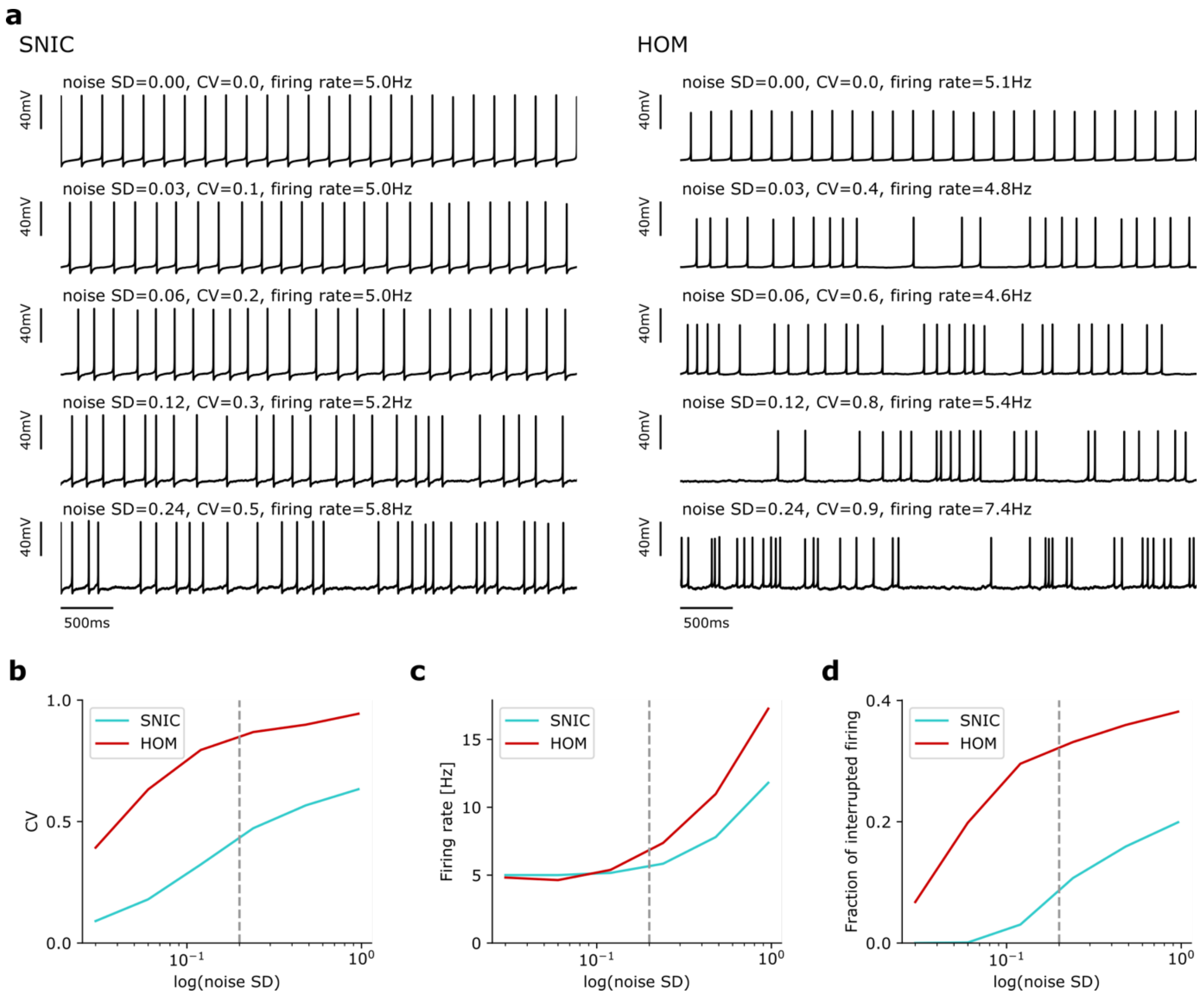

Fig. S6: Dependence of single cell characteristics on noise level. Noise SD in units of $\mu A\sqrt{s}/cm^2$. **a** Representative voltage traces for the Wang-Buzsáki model with different levels of stimulus noise at low temperature (SNIC on the left) and at high temperature (HOM on the right). **b** For all noise levels, the CV of the HOM neuron was higher compared to the SNIC neuron. **c** Firing rates started to increase compared to the baseline firing rate of 5 Hz for strong noise stimuli above 0.2 $\mu A\sqrt{s}/cm^2$, marked as dashed vertical line. **d** Bistability is reflected in high values of the fraction of pauses firing for the HOM neuron for noise inputs below 0.2 $\mu A\sqrt{s}/cm^2$ (marked as dashed vertical line), which do not strongly affect firing rates. Noise SD is divided by 1 $\mu A\sqrt{s}/cm^2$ before taking the logarithm.

4.3 Dependence of PRC measurements on noise

With an appropriate stimulus, PRC measurements are relatively stable against intrinsic noise, see also.[18] Even for intrinsic noise on the same amplitude as the noise stimulus, the PRC measurements deviate only slightly from the mathematical PRC of the model, as visible by a similar locking range in Fig. S7a compared to the measurement without intrinsic noise. Temporal correlations in the noise slightly weaken the increase of the locking range, see Fig. S7b-c.

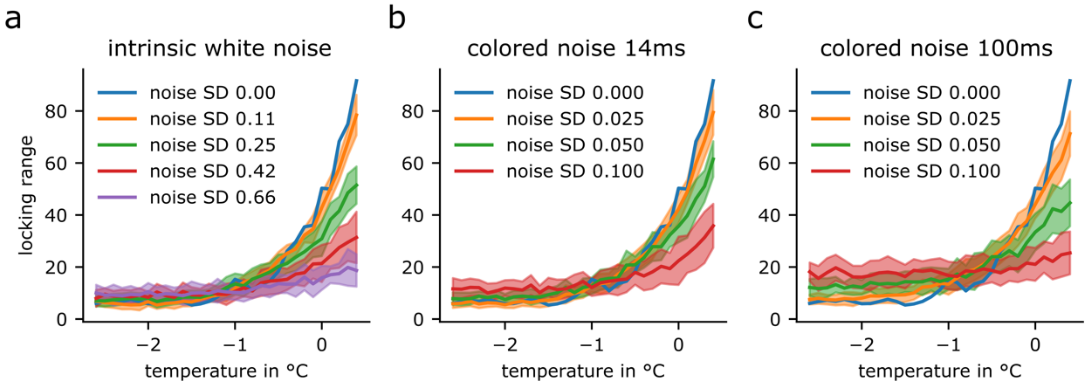

Fig. S7: Dependence of PRC measurements on internal noise. An external noise stimulus of 0.25 μA/cm^2 was used to probe the phase response of a Wang-Buzsáki model neuron at different temperatures, in analogy to the electrophysiological experiments. The model neuron additionally included an intrinsic noise source of different amplitudes and colors. **a** Locking range for different strengths of intrinsic noise that is white and Gaussian; noise standard deviations (SD) of 0, 0.11, 0.25, 0.42, 0.66 $\sqrt{s}\mu$A/cm^2, respectively. The stronger the intrinsic noise, the shallower the change in locking range when entering the HOM regime. At an SD of 0.25 $\sqrt{s}\mu$A/cm^2 the effect of the intrinsic noise on spiking is comparable or higher than that of the external noise (i.e., intrinsic and stimulus noise both result in a CV of 0.2 at -2.6 °C). For this level the measured locking range still shows a clear increase. For larger levels of intrinsic noise, the locking range decreases due to a continuous deterioration of the measured PRC, see[18] for details on the influence of intrinsic noise on PRC measurements. **b** Same for a colored intrinsic noise defined by an Ornstein-Uhlenbeck process with a time constant of 14 ms and intrinsic noise SD of 0, 0.025, 0.05 and 0.1 $\sqrt{s}\mu$A/cm^2, respectively. **c** Same for a colored intrinsic noise defined by an Ornstein-Uhlenbeck process with a time constant of 100 ms and intrinsic noise SD of 0, 0.025, 0.05 and 0.1 μA/cm^2, respectively. The time constant of external noise corresponds to one inter-spike interval of the noise-free neuron model. The increase in the temporal correlation of the noise results in a small reduction in the observed increase of locking range when approaching the HOM regime (compare corresponding traces in b and c). In all panels, the curves depict mean (solid line) and SD (borders of the shaded area) of 20 independent measurements of the PRC at the given noise level. Each measurement is based on a recording of 150 seconds length with the same external noise stimulus yet different intrinsic noise realizations.

4.4 Switch to homoclinic firing with small temperature changes

From our theoretical analysis, we expect that the critical temperature, at which the transition from SNIC to HOM occurs, is highly dependent on neuronal parameters, and thus likely different for most neurons. In order to capture as many transitions as possible, we have used a large temperature change in the experiments. Due to the finite life time of a patched cell, we decided to measure most cells only at the most extreme temperatures. However, for those neurons for which we looked at intermediate temperatures, the transition occurred also in the experiments suddenly. Supplementary Fig. S8 shows one exemplary cell, for which intermediate temperatures were recorded, and, as expected from the theory, the switch in neuronal firing occurs within a step of two degrees, from 35.8 °C to 37.7 °C. As a note of caution, the instantaneous switch in neuronal properties is a theoretical concept, for finite firing rates the effect will slightly wash out.

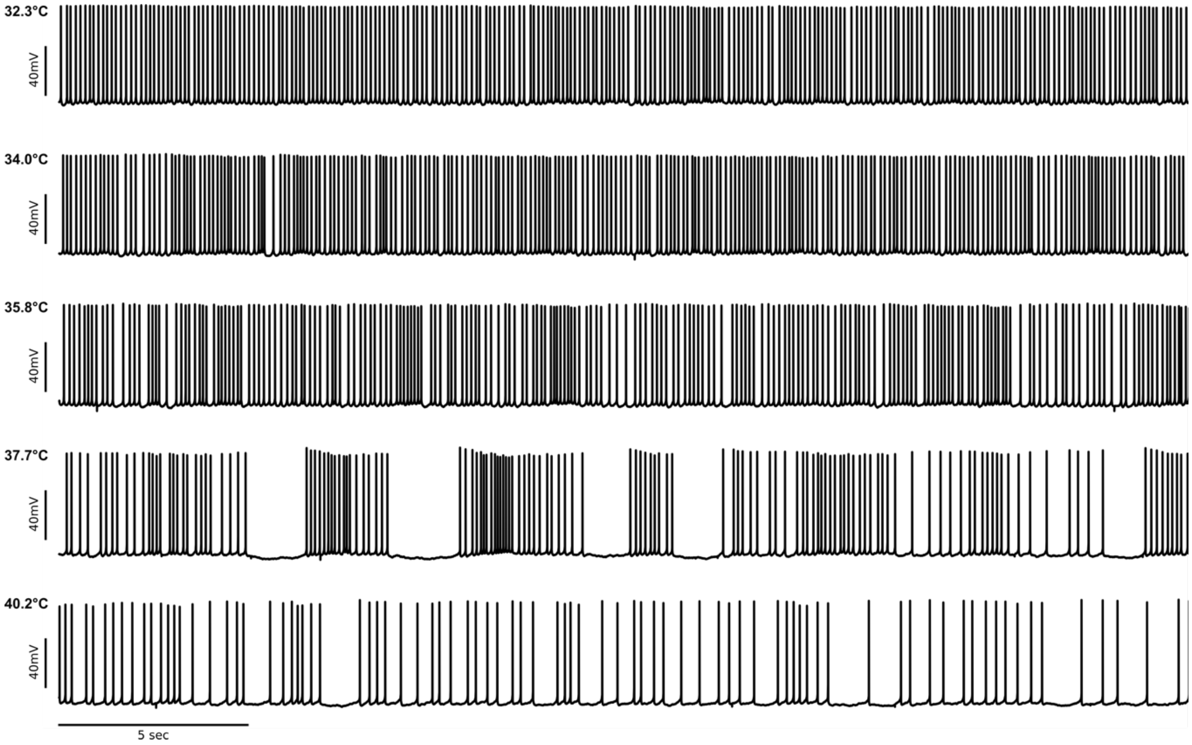

Fig. S8: CA1 pyramidal cell stimulated with direct current (DC) plus moderate noise at different temperatures. Shown is a 30 second trace out of a recording of one minute length. The switch occurs between 35.8°C and 37.7°C, in both recordings the same DC and noise gain are applied. The voltage trace at 35.8°C shows relatively regular spiking, as expected from a SNIC neuron. At 37.7 °C, the occurrence of long periods without spikes suggests a bistability between spiking and rest, consistent with homoclinic firing dynamics. The trace at 40.2 °C has a lower firing rate and is hence not directly comparable, yet the long silent periods still hint at a bistability and thus homoclinic firing dynamics.

5. Reversibility of the network effect

The network shown in Fig. 4 of the main text desynchronizes following a return to the low temperature. The desynchronization is slower than the synchronization, as shown in Fig. S9.

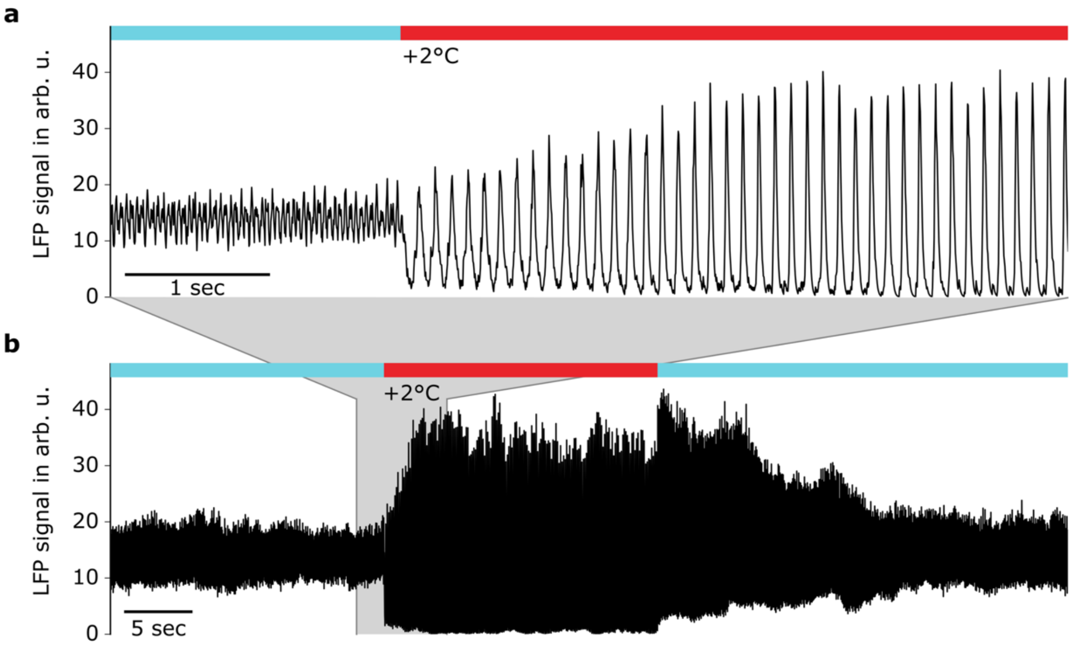

Fig. S9: The enhancing effect of temperature on network synchronization is reversible. Network synchronization (estimated by the LFP) increases with temperature elevation by 2 °C and decreases again when temperature is lowered. The timescale of change in synchronization differs: the timescale of synchronization onset with the temperature rise is faster than the desynchronization effect when temperature is reduced. The top panel presents a zoom in of the longer trace in the bottom panel; all details of the network simulation as in Fig. 4e in the main text.

Supplementary references

1. Izhikevich, E. M. *Dynamical Systems in Neuroscience*. (MIT Press, 2007).
2. Hesse, J., Schleimer, J.-H. & Schreiber, S. Qualitative changes in phase-response curve and synchronization at the saddle-node loop bifurcation. *Physical Review E* **95**, 052203 (2017).
3. Kirst, C., Ammer, J., Felmy, F., Herz, A. & Stemmler, M. Fundamental Structure and Modulation of Neuronal Excitability: Synaptic Control of Coding, Resonance, and Network Synchronization. *bioRxiv* 022475 (2015).
4. Contreras, S. A., Schleimer, J.-H., Gulledge, A. T. & Schreiber, S. Activity-mediated accumulation of potassium induces a switch in firing pattern and neuronal excitability type. *PLoS Computational Biology* **17**, e1008510 (2021).
5. Dubé, C. M., Brewster, A. L., Richichi, C., Zha, Q. & Baram, T. Z. Fever, febrile seizures and epilepsy. *Trends in neurosciences* **30**, 490–496 (2007).
6. Cross, J. H. Fever and fever-related epilepsies. *Epilepsia* **53**, 3–8 (2012).
7. Schuchmann, S. *et al.* Experimental febrile seizures are precipitated by a hyperthermia-induced respiratory alkalosis. *Nature Medicine* **12**, 817–823 (2006).
8. Schuchmann, S. *et al.* Respiratory alkalosis in children with febrile seizures. *Epilepsia* **52**, 1949–1955 (2011).
9. Tombaugh, G. C. & Somjen, G. G. Effects of extracellular pH on voltage-gated Na+, K+ and Ca2+ currents in isolated rat CA1 neurons. *The Journal of Physiology* **493**, 719–732 (1996).
10. Hille, B. Charges and potentials at the nerve surface. Divalent ions and pH. *The Journal of General Physiology* **51**, 221–236 (1968).
11. Otis, T. S. & Mody, I. Modulation of decay kinetics and frequency of GABAA receptor-mediated spontaneous inhibitory postsynaptic currents in hippocampal neurons. *Neuroscience* **49**, 13–32 (1992).
12. Banks, M. I., Li, T.-B. & Pearce, R. A. The Synaptic Basis of GABAA,slow. *Journal of Neuroscience* **18**, 1305–1317 (1998).
13. Huntsman, M. M. & Huguenard, J. R. Nucleus-Specific Differences in GABAA-Receptor-Mediated Inhibition Are Enhanced During Thalamic Development. *Journal of Neurophysiology* **83**, 350–358 (2000).
