# Supplementary material for: Temperature elevations can induce switches to homoclinic action potentials that alter neural encoding and synchronization
Source: Nat Commun. 2022 Jul 8;13:3934. doi: 10.1038/s41467-022-31195-6 (PMC9270341; doi:10.1038/s41467-022-31195-6)
Supplement: Supplementary file 2 — Reporting Summary [file 41467_2022_31195_MOESM2_ESM.pdf]

nature portfolio

Corresponding author(s): Susanne Schreiber
Last updated by author(s): Jun 2, 2022

Reporting Summary

Nature Portfolio wishes to improve the reproducibility of the work that we publish. This form provides structure for consistency and transparency in reporting. For further information on Nature Portfolio policies, see our Editorial Policies and the Editorial Policy Checklist.

Statistics

For all statistical analyses, confirm that the following items are present in the figure legend, table legend, main text, or Methods section.

n/a	Confirmed	
☐	☒	The exact sample size (*n*) for each experimental group/condition, given as a discrete number and unit of measurement
☐	☒	A statement on whether measurements were taken from distinct samples or whether the same sample was measured repeatedly
☐	☒	The statistical test(s) used AND whether they are one- or two-sided<br>*Only common tests should be described solely by name; describe more complex techniques in the Methods section.*
☒	☐	A description of all covariates tested
☒	☐	A description of any assumptions or corrections, such as tests of normality and adjustment for multiple comparisons
☐	☒	A full description of the statistical parameters including central tendency (e.g. means) or other basic estimates (e.g. regression coefficient) AND variation (e.g. standard deviation) or associated estimates of uncertainty (e.g. confidence intervals)
☐	☒	For null hypothesis testing, the test statistic (e.g. *F*, *t*, *r*) with confidence intervals, effect sizes, degrees of freedom and *P* value noted<br>*Give P values as exact values whenever suitable.*
☒	☐	For Bayesian analysis, information on the choice of priors and Markov chain Monte Carlo settings
☒	☐	For hierarchical and complex designs, identification of the appropriate level for tests and full reporting of outcomes
☒	☐	Estimates of effect sizes (e.g. Cohen's *d*, Pearson's *r*), indicating how they were calculated

Our web collection on statistics for biologists contains articles on many of the points above.

Software and code

Policy information about availability of computer code

Data collection	analogue-to-digital converter Digidata 1550A (Molecular Devices), and Clampex Software (version 10.7) in PClamp Software Suite, Molecular Devices
Data analysis	python (version 2.7.16), brian2 (version 2.3.0.2), spykeviewer (0.4.1), inkscape (version 1.1), matlab (version 9.7.0.1190202), AUTO-07P (version 0.7)

For manuscripts utilizing custom algorithms or software that are central to the research but not yet described in published literature, software must be made available to editors and reviewers. We strongly encourage code deposition in a community repository (e.g. GitHub). See the Nature Portfolio guidelines for submitting code & software for further information.

Data

Policy information about availability of data

All manuscripts must include a data availability statement. This statement should provide the following information, where applicable:
- Accession codes, unique identifiers, or web links for publicly available datasets
- A description of any restrictions on data availability
- For clinical datasets or third party data, please ensure that the statement adheres to our policy

The experimental data generated in this study have been deposited at zenodo.org under DOI: 10.5281/zenodo.6589677.

Field-specific reporting

Please select the one below that is the best fit for your research. If you are not sure, read the appropriate sections before making your selection.

☒ Life sciences ☐ Behavioural & social sciences ☐ Ecological, evolutionary & environmental sciences

For a reference copy of the document with all sections, see nature.com/documents/nr-reporting-summary-flat.pdf

Life sciences study design

All studies must disclose on these points even when the disclosure is negative.

Sample size	N=38
Data exclusions	Only N=3 cells fulfilled the prerequisites (reliable PRC estimate, SNIC firing mode). Data of these cells are shown; two of which displayed the effect discussed in the paper, one of which did not.
Replication	The effect was replicated in two cells.
Randomization	For the proof of principle approach randomization is not applicable.
Blinding	The explorative nature of the study design does neither require binding nor is it possible.

Reporting for specific materials, systems and methods

We require information from authors about some types of materials, experimental systems and methods used in many studies. Here, indicate whether each material, system or method listed is relevant to your study. If you are not sure if a list item applies to your research, read the appropriate section before selecting a response.

Materials & experimental systems

n/a	Involved in the study	
☒	☐	Antibodies
☒	☐	Eukaryotic cell lines
☒	☐	Palaeontology and archaeology
☐	☒	Animals and other organisms
☒	☐	Human research participants
☒	☐	Clinical data
☒	☐	Dual use research of concern

Methods

n/a	Involved in the study	
☒	☐	ChIP-seq
☒	☐	Flow cytometry
☒	☐	MRI-based neuroimaging

Animals and other organisms

Policy information about studies involving animals; ARRIVE guidelines recommended for reporting animal research

Laboratory animals	Mus musculus, C57Bl/6/N, male, age 10-13 days and 22-30 days
Wild animals	This study did not involve wild animals.
Field-collected samples	This study did not involve field-collected samples.
Ethics oversight	Animal maintenance and experiments were in accordance with the guidelines of local authorities (Berlin state government, T0100/03), and they followed the German animal welfare act and the European Council Directive 2010/63/EU on protection of animals used for experimental and other scientific purposes.

Note that full information on the approval of the study protocol must also be provided in the manuscript.
